# The effects of caffeine and d-amphetamine on spatial span task in healthy participants

**DOI:** 10.1371/journal.pone.0287538

**Published:** 2023-07-13

**Authors:** Faiz M. Kassim, J. H. Mark Lim, Sophie V. Slawik, Katharina Gaus, Benjamin Peters, Joseph W. Y. Lee, Emily K. Hepple, Jennifer Rodger, Matthew A. Albrecht, Mathew T. Martin-Iverson

**Affiliations:** 1 Psychopharmacology Research Unit, School of Biomedical Sciences, University of Western Australia, Perth, WA, Australia; 2 Department of Psychiatry, St. Paul’s Hospital Millennium Medical College, Addis Ababa, Ethiopia; 3 Faculty of Human and Health Sciences, Psychology, University of Bremen, Bremen, Germany; 4 Psychiatry, Medical School, University of Western Australia, Perth, WA, Australia; 5 Mental Health, North Metropolitan Health Services, Perth, WA, Australia; 6 Experimental and Regenerative Neurosciences, School of Biological Sciences, University of Western Australia, Crawley, WA, Australia; 7 Brain Plasticity Group, Perron Institute for Neurological and Translational Science, Nedlands, WA, Australia; 8 Western Australian Centre for Road Safety Research, School of Psychological Science, University of Western Australa, Crawley, WA, Australia; PLoS ONE, UNITED STATES

## Abstract

Studies that examined the effect of amphetamine or caffeine on spatial working memory (SWM) and verbal working memory (VWM) have used various tasks. However, there are no studies that have used spatial span tasks (SSTs) to assess the SWM effect of amphetamine and caffeine, although some studies have used digit span tasks (DST) to assess VWM. Previous reports also showed that increasing dopamine increases psychosis-like experiences (PLE, or schizotypy) scores which are in turn negatively associated with WM performance in people with high schizotypy and people with schizophrenia. Therefore, the present study aimed to examine the influence of d-amphetamine (0.45 mg/kg, PO), a dopamine releasing stimulant, on SST, DST, and on PLE in healthy volunteers. In a separate study, we examined the effect of caffeine, a nonspecific adenosine receptor antagonist with stimulant properties, on similar tasks. **Methods:** Healthy participants (N = 40) took part in two randomized, double-blind, counter-balanced placebo-controlled cross-over pilot studies: The first group (N = 20) with d-amphetamine (0.45 mg/kg, PO) and the second group (N = 20) with caffeine (200 mg, PO). Spatial span and digit span were examined under four delay conditions (0, 2, 4, 8 s). PLE were assessed using several scales measuring various aspects of psychosis and schizotypy. **Results:** We failed to find an effect of d-amphetamine or caffeine on SWM or VWM, relative to placebo. However, d-amphetamine increased a composite score of psychosis-like experiences (p = 0.0005), specifically: Scores on Brief Psychiatric Rating Scale, Perceptual Aberrations Scale, and Magical Ideation Scale were increased following d-amphetamine. The degree of change in PLE following d-amphetamine negatively and significantly correlated with changes in SWM, mainly at the longest delay condition of 8 s (r = -0.58, p = 0.006). **Conclusion:** The present results showed that moderate-high dose of d-amphetamine and moderate dose of caffeine do not directly affect performances on DST or SST. However, the results indicate that d-amphetamine indirectly influences SWM, through its effect on psychosis-like experiences.

**Trial registration. Clinical Trial Registration Number:** CT-2018-CTN-02561 (Therapeutic Goods Administration Clinical Trial Registry) and ACTRN12618001292268 (The Australian New Zealand Clinical Trials Registry) for caffeine study, and ACTRN12608000610336 for d-amphetamine study.

## Introduction

Non-medical use of psychostimulants is common in healthy subjects, often for enhancing cognitive performance in domains such as memory or vigilance ([see 1 for review, 2–4]). Several studies have shown that amphetamine improves subjective ratings of arousal and perceived enhancements [5], and objective measures of cognitive performance in sleep-deprived participants [6–11]. However, the cognitive enhancing effects of amphetamine healthy subjects are mixed and more inconclusive on the general domain cognition [12].

Inconsistent results have been found for amphetamine on working memory (WM) [5,13–19] and executive function or performance speed [6,13,18,20–26]. Studies that examined the WM effects of amphetamine used various types of spatial tasks [5,6,16,17,19,22,27–29] and limited types of verbal tasks [14,15,18,25,30,31]. All of the studies (except one [31]) that used digit span tasks (DST) to examine verbal WM (VWM) reported that d-amphetamine did not affect performances on DST. The findings indicate that DST is not a sensitive tool to detect the effects of d-AMP on WM, ([for review, see 12]). In addition, there were no differences in effects of d-amphetamine that used either forward, backward or both tasks of the DS, which again implies that the recall direction is not sensitive to detect the effects of d-amphetamine on WM [32]. In contrast, studies that examined spatial WM (SWM) used various spatial tasks and some of them found drug and time interactional effects or main effects of AMP. However, there are no studies that used spatial span tasks (SST) to assess the SWM effect of AMP.

Caffeine is also a psychostimulant that is widely consumed worldwide for its psychomotor and cognitive effects. Although caffeine has less strong pharmacological effects than other strong psychostimulants (such as amphetamines), the qualitative physiological effects of caffeine are expected to be broadly similar as the other stimulants [33,34]. Like amphetamine, caffeine increases subjective ratings of arousal [35] and objective measures of cognitive performance in sleep-deprived participants [6–11]. However, although findings are consistent for some domains, the direct cognitive enhancing effects of caffeine has not been well-established for broader domain [36,37]. Regarding the acute effects of caffeine on WM, studies indicated that caffeine has no effect on visuo-spatial WM [38] and VWM in healthy people [39–41]. Importantly, the findings showed that acute caffeine administration consistently enhances information processing, attention, vigilance or reaction time regardless of the cognitive task type [36,37], without necessarily changing WM performance accuracy [42]. A review of 24 studies testing attention concluded that acute caffeine consumption consistently enhances attention on simple and complex tasks [43]. However,the acute effects of caffeine on the broader spectrum of neuropsychological domains is inconsistent [37,39–41,44–54] For instance, some of the studies reported that caffeine enhances cognitive performance on digit symbol substitution (DSST), cancellation task, Stroop test, or auditory and visual recall [39,44,46,48,51,52]., Other studies that tested other cognitive domains demonstrated that caffeine does not affect performances on the DSST, the Stroop test, and the four part continuous performance test [52–54] Based on the effects of caffeine on limited cognition domains, researchers suggest that caffeine is not “a pure” cognitive enhancer but indirectly enhances cognition by increasing performance under suboptimal condition such as fatigue state [36]. Overall, the reports indicate that acute consumption of caffeine has positive effects on vigilance, but fails to enhance performance (accuracy) on higher-order cognitive tasks. However, like amphetamine, there are no studies that assessed the SWM effect of caffeine using SST.

A main challenge in the assessment of WM performance is that that there is no one generally accepted SWM or VWM model [55]. Therefore, different studies have used different tasks to measure SWM. Furthermore, individual differences in personality traits such as neuroticism and schizotypy are important factors that may also cause performance differences on WM tasks among individuals ([for detail, see 56,57]). It has been suggested that dopamine may play the linking role between schizotypy/PLE and WM performance [12]. The prefrontal cortex (PFC), particularly the dorsolateral (DLPFC), plays a critical role in SWM in healthy individuals and people with schizophrenia ([for review, see 58–63]). Specifically, studies in non-human primates [64–69], rodents [63], ADHD individuals [70] and people with schizophrenia [71,72] have demonstrated that the optimal level of dopamine in the PFC have a crucial role for SWM performance ([see 73 for review, 74]). Studies also showed that increasing dopamine increases PLE scores and the level of dopamine released positively correlates with PLE scores [75]. For instance, the released amount of dopamine in striatal and extra-striatal regions was found to correlate with PLE scores after d-amphetamine administration (0.43 mg/kg, PO) in sixty-three subjects [76]. Interestingly, the dopamine release level after d-amphetamine administration in high schizotypy participants was intermediate between healthy control groups and acutely ill psychotic patients [77].

Caffeine also modulate the dopaminergic system indirectly through adenosine-dopamine receptor heteromer (A2A-D2) [34,78–80], which is one potential mechanism to influence WM. The influence of personality trait has been also extended to the acute effect of caffeine on WM [49,50]. For instance, caffeine enhanced WM performance in extrovert participants [50], although it failed to improve WM in the overall participants in this study [50] and other studies [38–41]. Therefore, pharmacological manipulation of psychosis or PLE/Schizotypy will have a double advantage: first, the model enables to characterise the neurochemical mechanism of the disease onset and progress; second, it may better tell the degree a PLE induced by the administered drug (i.e., psychotomimetic effect) is influenced by the baseline scores. In general, both d-amphetamine and caffeine are psychostimulants that increase dopamine signalling in the human brain and are consumed by healthy subjects to enhance cognitive performance. However, there are limited numbers of studies that focused on the higher-order cognitive effects of caffeine, to compare with dexamphetamine [37]. Specifically, there are no studies that assessed the WM effects of each drug using SST. Previous studies such as Loke and colleagues recommended comparing the behavioural/cognitive effects of caffeine (the mild stimulant) and amphetamine (the strong stimulant) to elucidate the magnitude of dexamphetamine or caffeine’s effect [46]. Therefore, we designed two randomised studies on the WM effects of dexamphetamine and caffeine. In experiment 1, we aimed to study the effect of moderate to high dose of d-amphetamine (0.45 mg/kg, PO) on SWM performance in healthy subjects using SST, under delay conditions. In experiment 2, we aimed to study the effect of a moderate dose of caffeine (200 mg) on SWM. The study also aimed to examine the effects of each drug on VWM using DST to identify whether the effects of d-amphetamine and caffeine are specific to SST in the presence of delay conditions (i.e., the DST is a control condition that involves non-SWM and is included as a control task). As some of the previous studies showed that d-amphetamine improves SWM but not VWM and given that d-amphetamine is a stronger psychostimulant than caffeine, we predicted that d-amphetamine but not caffeine enhances performance on SST in the presence of delay conditions.

## Methods and materials

### Ethical approval

The University of Western Australia (UWA) Human Research Ethics Committee has granted ethical approval for this study (RA/4/1/8056 for experiment 1 and RA/4/20/4558 for experiment 2). The study is registered at the Australian New Zealand Clinical Trials Registry (ACTRN12608000610336, for experiment 1 and ACTRN12618001292268, for experiment 2).

### Participant recruitment

All forty participants were recruited by word of mouth, lecture announcements, advertisements on campus notice boards, social media, or university-group emails in 2017 and 2018. Participants were informed to abstain from any psychoactive substance (including caffeine) at least 24 hours before the test.

Inclusion criteria were being >17 and <60 years of age, and if female, not be pregnant, and be using contraceptives if sexually active and fertile to avoid the potential of harming a fetus. Exclusion criteria included, inability to provide valid consent, sensitivity to d-amphetamine or sympathomimetic amines for the d-amphetamine study or to caffeine in the caffeine study, history of psychotic disorders (schizophrenia, schizophrenia-spectrum, or bipolar affective disorder) in themselves or their first-degree relatives, substance-use dependence, neurological illness, or language difficulties that could interfere with assessment, currently taking any (prescription) medicine that affect cognitive functions, childhood diagnosis of ADHD, women who are pregnant or lactating and cardiovascular disorder risks.

The testing sessions were held between 9:00 am to 5:00 pm for a total of two days (with one-week separation) for each participant. As mentioned in the general procedures, the times of the experiments at each session and test were well-controlled to avoid inconsistencies. Transport and lunch were provided with no additional financial incentive. Informed consent and medical assessments by psychiatrists were conducted before the experiment on the first day. The basic demographic information (age, years of education, sex, and weight) was then taken.

**Fig 1:**
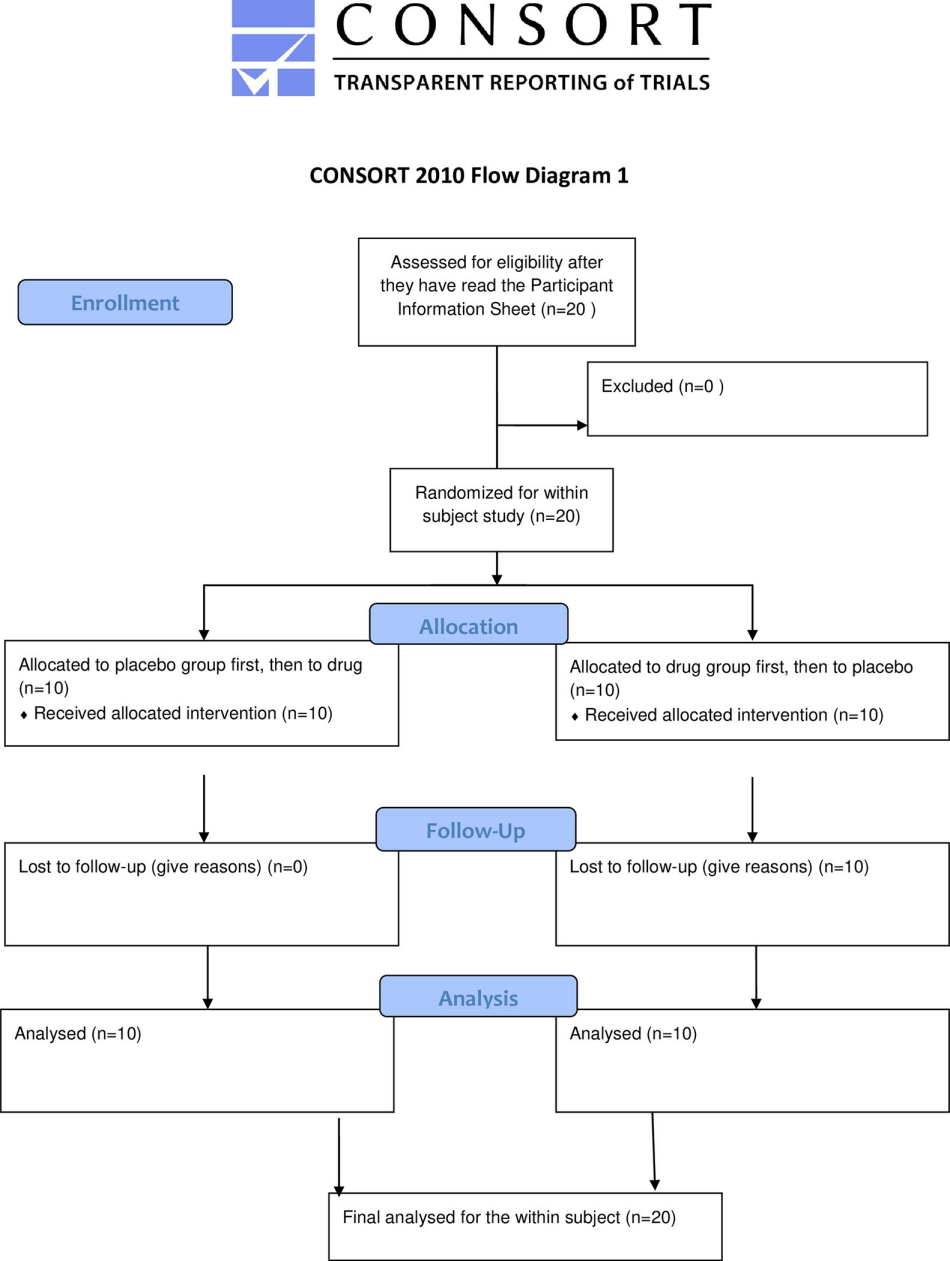
CONSORT 2010 Flow Diagram 1.

**Fig 2:**
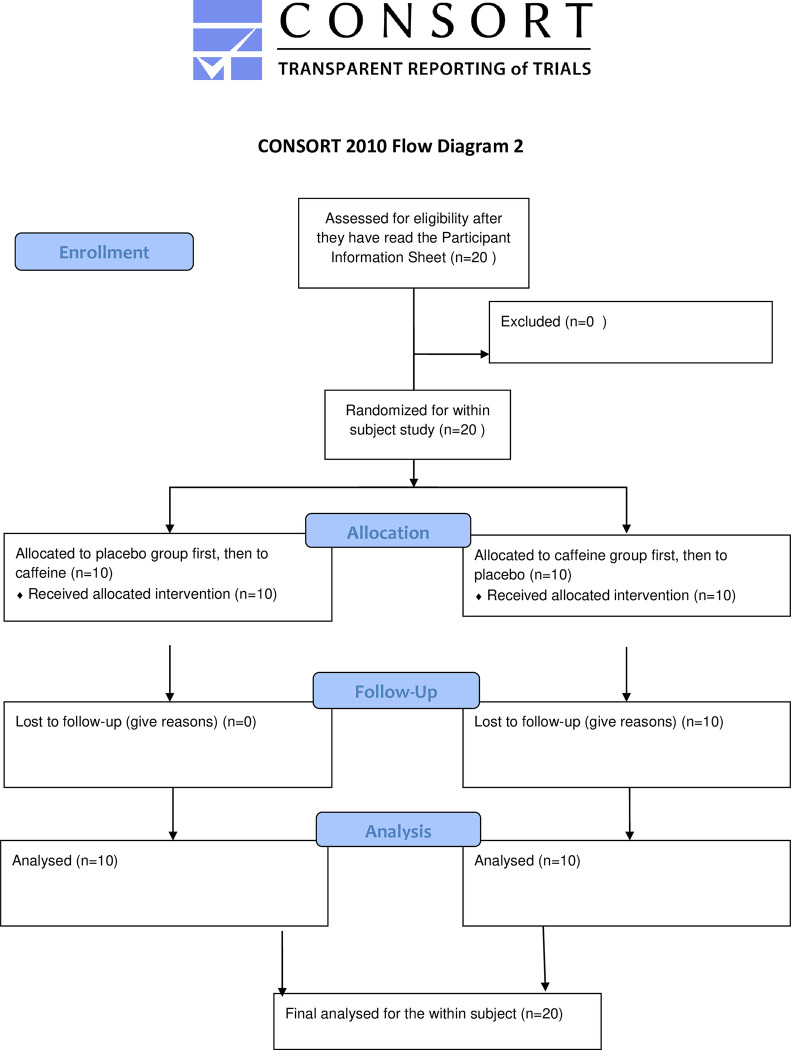
CONSORT 2010 Flow Diagram 2.

Both experiments were randomized, double-blind, counter-balanced, placebo controlled cross-over studies with permuted block randomization for drug order. Each participant received both the active drug and placebo in identical gelatin capsules. Half of the participants (n = 10) in each experiment received placebo on the first day and active drug on the second day which followed the first day by at least a week, while the remaining half received active drug first and placebo second (Figs 1 and 2).

The principal investigator generated the random allocation sequence, allocated participants to each group and only he had access to information that could identify individual participants during or after data collection. The trial staff that were involved in the participant recruitment and data collection did not have the randomization list.

### Experiment 1: D-amphetamine

#### Participants

Twenty healthy participants (15 male) with a mean (±SD) age of 22.9 (±5.17) were recruited. They had a mean weight (± SD) of 74.9 (± 14.2) kg and 14.5 (±1.5) years of education. Substance use information was unavailable for one participant: 13 of the participants were regular consumers of caffeinated drinks; 8 of them had used cannabis at least once (6 of them used it in the last two weeks); 11 of them consumed amphetamine at least once in their life (3 of them consumed in the last one year, while 2 of them consumed it in the last three months). None of them were active cigarette smokers (only three of them smoked a month ago).

#### Drug and design

D-amphetamine (0.45 mg/kg, PO, Aspen Pharmacare, Australia) was used, giving a mean dosage of 33.7 mg based on the mean weight of the participants of 74.9 kg. Similar sizes and numbers of capsules containing either placebo (glucose) or 0.45 mg/kg d-amphetamine were prepared using 1, 2.5, 5, and 10 mg d-amphetamine sulfate tablets. This dose of d-amphetamine was selected based on previous experiments in healthy subjects demonstrating significant effects on a range of illusory and psychophysiological measures [15,76,81,82].

### Experiment 2: Caffeine

#### Participants

Twenty healthy participants (14 males) with the mean (±SD) age of 22.2 (± 4.1) were recruited. They had a mean weight (± SD) of 80.3 kg (± 20.0) and 14.6 (±1.5) years of education. All except three were a regular consumer of caffeine (have taken at least one cup of caffeine-containing drinks per day, with 1.6 (±1.1) mean (±SD) cup of coffee or tea). Three of them consumed cannabis in the last two months, four of them smoke cigarette and two of them consumed amphetamine in the last month.

#### Drug and design

For this experiment, 200 mg of caffeine was administered in the morning (PO, BID; caffeine 200mg, PROLAB nutrition LLC, Chatsworth, USA). Caffeine was administered in gelatin capsules (size 0). Powdered glucose (Placebo) was also separately placed in the same type of capsule. This dose of caffeine was selected based on previous studies on cognitive tasks [41,83,84] at 200 mg.

### General procedures for both experiments

#### Psychological scale

Five rating scales were conducted once each day to measure Psychosis-like experiences (PLE): Brief Psychiatric Rating Scale (BPRS) [85], Scale for the Assessment of Positive Symptoms (SAPS) [86], Perceptual Aberrations Scale (PAS) [87], Revised Launay-Slade Hallucinations Scale (LSHS-R) [88] and Magical Ideation Scale (MIS) [89]. Three were self-report questionnaires and two were interview-based. The measurement times were at 120 min post- d-amphetamine administration and at 30 min post-caffeine administration.

#### Physiological measures

Physiological changes were measured to assess whether the drug was active or not during the WM tests. Blood pressure (BP), temporal vein temperature (Temp) and heart rate (HR) were measured in triplicate five times each day to follow the time course of the drug and placebo effects on Systolic BP (SBP), Diastolic BP (DBP), HR, and Temp. BP (mmHg) and HR [in beat per minute (bpm)] were measured. The consecutive five physiological testing times were 0, 60, 110, 210, and 370 min post-d-amphetamine administration, while 0, 60, 210, 300, and 390 min post-caffeine administration.

#### Working memory

WM tests were conducted in a well-lit room. We used forward spatial span (for SWM) and digit span (for VWM), from the Wechsler Adult Intelligence III (WAIS-III) [90]. The WM tests post-d-amphetamine administration were conducted at 90 and 210 min. We chose this time interval for d-amphetamine as previous studies showed that peak plasma level of oral amphetamine occurs between 90–120 to 180–210 min [27,91–93] and behavioural effects would be seen about 30 min of post-administration [94]. The WM tests post-caffeine administration were was conducted at 60 and 120 min, as previous studies showed that the peak plasma concentration of caffeine reaches between 15 and 120 minutes [95].

The same scoring criteria (the total scored and the maximum obtained) were used for both digit span and spatial span tasks. The maximum obtained indicates the maximum number of trials the participant scored without any error, while the total scored is the total number of trials correctly scored from the 16 trials until the participant fails the same item on subsequent trials. A measure of WM consisted of the average of the digit and spatial span scores across the delay conditions. Similar overall scores were calculated for SWM and VMW.

#### Spatial Span

Spatial span is a visuospatial analogue of the digit span test. This test was conducted at 90 min of post-d-amphetamine administration (0.45 mg/kg) and at 60 min post-caffeine administration. For each trial, ten randomly arranged blue squares (Psychcorp Mark) were shown on the table. The task began with the simplest level of a two-box sequence. After each successful trial, the number of boxes in the sequence was increased by one to a maximum of nine. There were eight items, each with two trials of the same box number (i.e., a total of 16 trials). There were four delay conditions (0, 4, 6, and 8 s delay): the participant was asked to touch the boxes in the same order (forward) right after the examiner (0 s delay), and repeated for 4, 6, and 8 s delays (but different compositions). In addition to the intrinsic differences among tasks, delay condition between encoding and maintenance phase might be a key factor in WM performance [96–98]. Previous studies found that impairment of SWM and VWM in people with schizophrenia spectrum disorder is intensive during delayed response tasks [e.g., 99]. Therefore, investigating SST and DST with delays is important to assess both immediate and delayed WM capacity and applying delays during WM tasks may increase the sensitivity of the tasks the effects of drugs such as d-amphetamine and caffeine.

#### Digit Span

This test was carried out after the spatial test, at 210 min of post-d-amphetamine administration and at 120 minutes of post-caffeine administration. The digits were pronounced in 1 s intervals by the examiner. The participant was asked to repeat the digits in the same order (forward) right after the examiner (0 s delay), and repeated for 4, 6, and 8 s delays (but different digit compositions). The task of recalling the digits began with the simplest level of a two-digit sequence. After each successful trial, the number of digits in the sequence was increased by one to a maximum of nine. There were eight items (the minimum item was with two digits, while the maximum was nine digits), two trials of the same length of digits for each item (i.e., a total of 16 trials).

#### Statistical analysis

The statistical analysis was performed using R programming version 3.5.3 (R Core Development Team 2018) and dply, ez, lme4, plyr, Rmisc, stats, sm packages. Normal Q-Q plots were used to check the residuals of the data. A repeated measure analysis (ANOVA) was used to analyse the data if the residuals indicated a sufficiently normal distribution. Then paired t-tests with exact Bonferroni corrections were used for pairwise comparisons between the drug and placebo condition. Wilcoxon signed rank test with continuity correction (and with Bonferroni corrections) was used if the residuals were not distributed normally. Subsequently, physiological measurements and WM were analysed using ANOVA with Greenhouse–Geisser epsilon if the assumption of sphericity was violated, and generalised η^2^ effect sizes were calculated. Linear mixed effect model was used to analyse the effects of session (i.e., day order) on WM. The scores for each PLE scale were converted to z-scores based on the Grand Mean and SD for that specific scale, and the total z-score for each scale was calculated. The PLE scores were calculated as the unweighted average of the total z-scores from all the psychological measures. Pearson’s correlation was used to analyse correlation. Exceptions to these methods are listed in the results.

#### Power analysis

We used G*Power 3.1 power calculator, and based our predicted mean difference (Drug-Placebo) and standard deviation of the difference on findings in our lab on the effect of DEX on the "embodiment" component of the RHI (paired samples t-test) indicating an effect size of 0.43 with alpha = 0.05, with a power of 0.80 (80%), with a two-tailed test.

## Results

### Experiment 1: D-amphetamine

#### D-amphetamine effects on physiology

There were significant effects of d-amphetamine on heart rate (F[1, 19] = 49.8, p < 0.001, ƞ^2^ = 0.18), systolic BP (F[1, 19] = 49.6, p < 0.0001, ƞ^2^ = 0.18) and diastolic BP [F(1, 19] = 35.7, p < 0.001, ƞ^2^ = 0.24).

#### D-amphetamine effects on VWM and SWM

ANOVA with delay and drug as within-subject factors and drug order as a between-subjects factor indicated that there were no drug by delay interactions on VWM (maximum obtained F[3, 57] = 0.5, p = 0.67, ƞ^2^ = 0.004; and total scored F[3, 57] = 0.67 p = 0.57, ƞ^2^ = 0.003) or SWM (maximum obtained F[3, 57] = 0.29, p = 0.82, ƞ^2^ = 0.006; and total scored F[3, 57] = 0.02, p = 0.94, ƞ^2^ = 0.0003).

**Fig 3:**
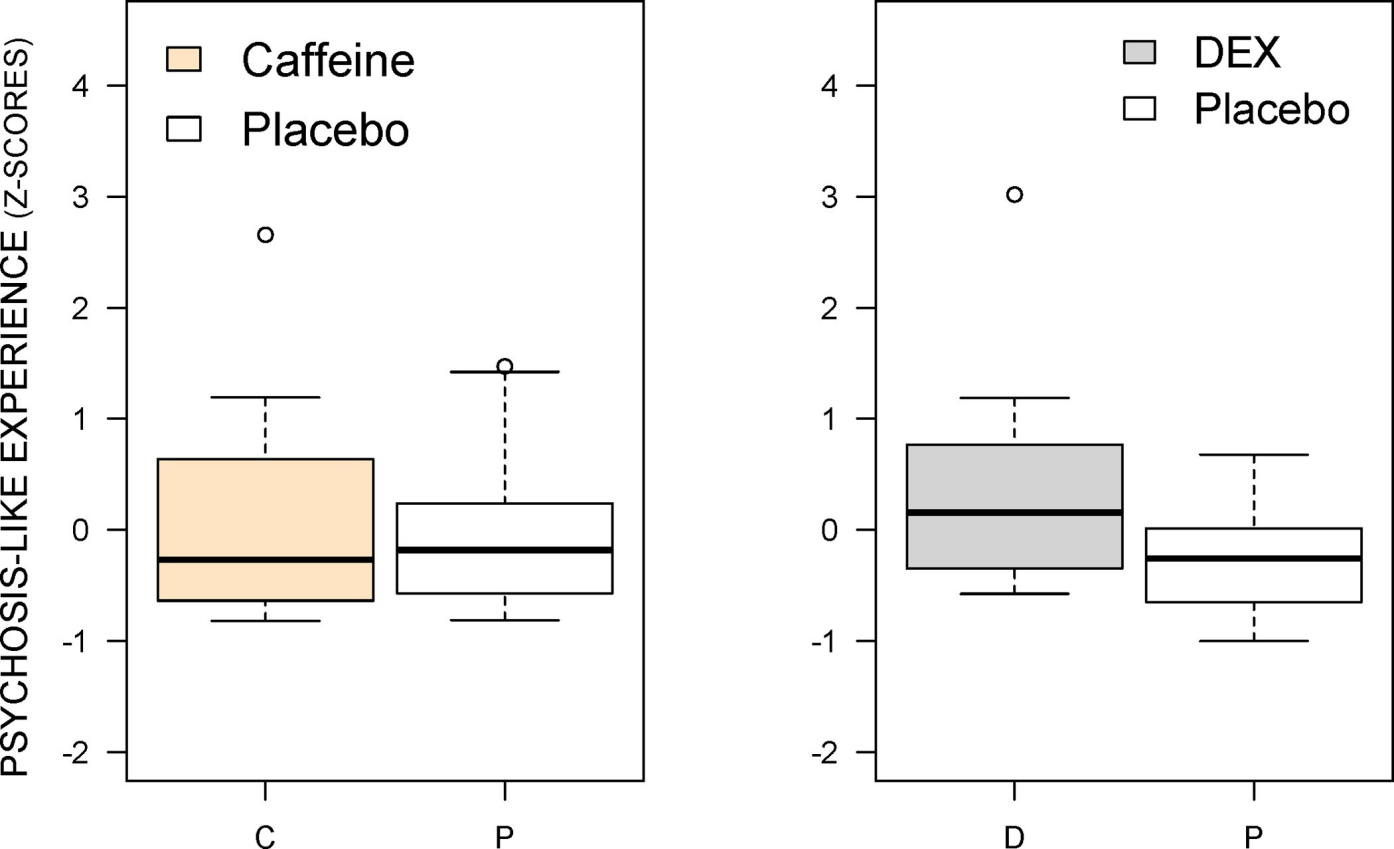
The effect of d-amphetamine (0.45 mg/kg, PO) and placebo on working memory (WM) for maximum obtained and total scored. Data are presented as maximum scored (A and C) or total scored (B and D) vs. delay conditions. A) The effect of d-amphetamine on spatial WM (maximum scored), relative to placebo; B) The effect of d-amphetamine on spatial WM (total scored), relative to placebo; C) The effect of d-amphetamine on verbal WM (maximum scored), relative to placebo; D) The effect of d-amphetamine on verbal WM (total scored), relative to placebo. There were no significant effects of d-amphetamine on WM, VWM or SWM.

As shown in Fig 3, there were no significant main effects of d-amphetamine on VWM (for the maximum obtained F[1, 19] = 0.27 p = 0.60, ƞ^2^ = 0.001; and total scored (F[1, 19] = 0.05, p = 0.8, ƞ^2^ = 0.0030) or SWM (for the maximum obtained F[1, 19] = 0.78, p = 0.38, ƞ2 = 0.003; and total scored (F[1, 19] = 1.6, p = 0.22, ƞ^2^ = 0.005). Linear mixed effects model (with Satterthwaite’s method) failed to find an interaction between drug and session on SWM (maximum obtained F[1, 18] = 0.01, p = 0.77; total obtained F[1, 18] = 0.18, p = 0.67) and VWM (maximum obtained F[1, 18] = 0.2, p = 0.63; total scored F[1, 18] = 1.6, p = 0.21). However, there was an effect of session on SWM (total obtained F[1, 72] = 5.1, p = 0.028) but not on SWM (maximum obtained F[1, 126] = 1.6, p = 0.20) and VWM (maximum obtained F[1, 18] = 3.4, p = 0.08; total scored F[1, 18] = 2.4, p = 0.13). The M±SD of SWM total scored during day 1 and day 2 are 9.5±1.7 and 9.9±.5, respectively.

There were significant effects of delay on VWM for the total scored (F[2.3, 43.9] = 22.3, p = 0.004, ƞ^2^ = 0.046, after correction with Greenhouse–Geisser epsilon) but not for maximum obtained (F[3, 57] = 1.4, p = 0.24, ƞ^2^ = 0.018). Likewise, there were significant delay effects on SWM for the total scored (F[2.8, 53] = 6.6, p = 0.0006, ƞ^2^ = 0.074, after correction with Greenhouse–Geisser epsilon) but not for the maximum obtained (F[3, 57] = 2.1, p = 0.11, ƞ^2^ = 0.03).

#### D-amphetamine effect on PLE

**Fig 4:**
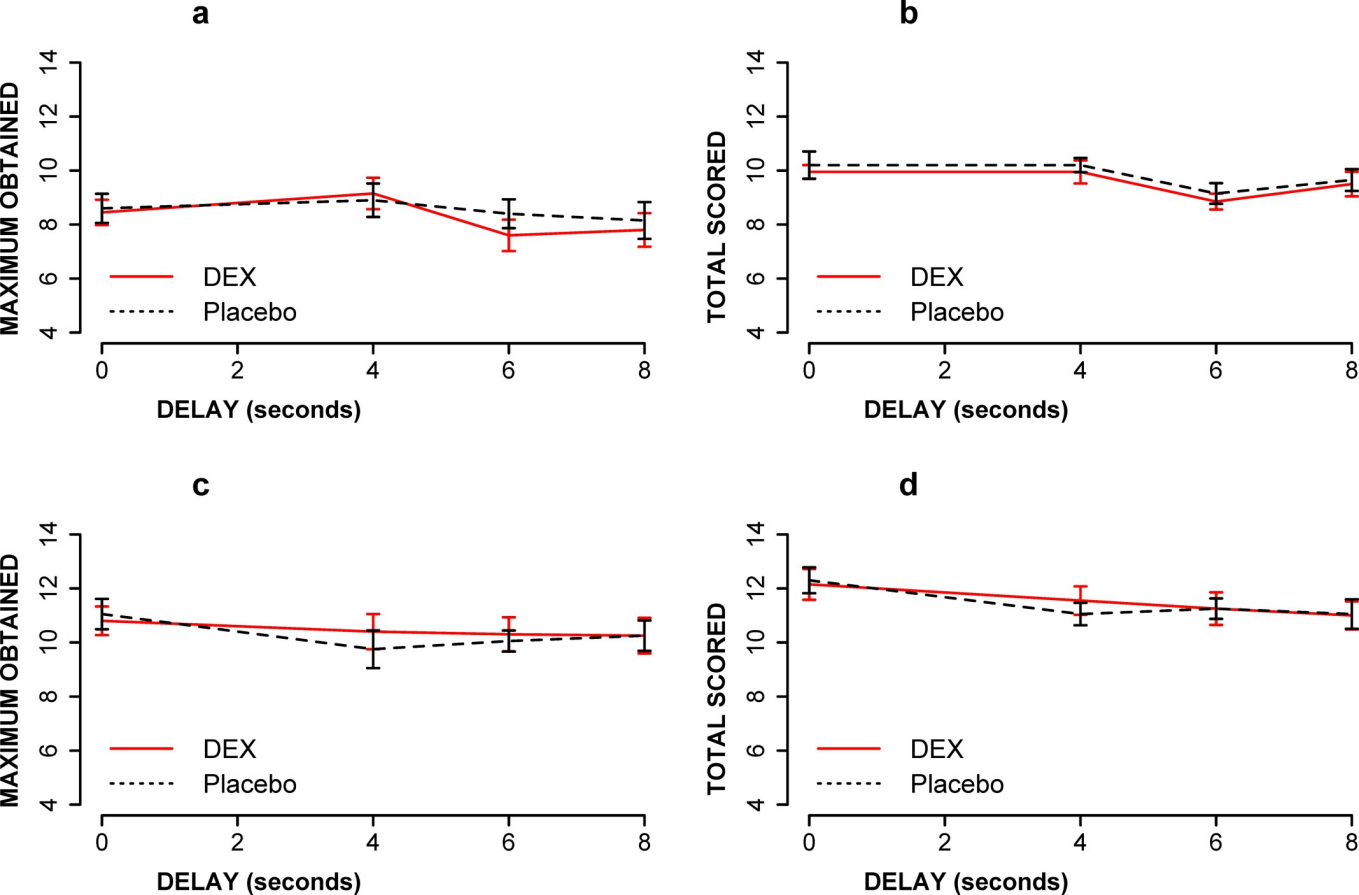
Box plot of the effect of d-amphetamine (0.45 mg/kg, PO) and caffeine on Psychosis-like experiences (PLE). D-amphetamine but not caffeine significantly increased PLE scores, relative to placebo (p < 0.001). C: Caffeine, D: D-amphetamine, P: Placebo.

As shown in Fig 4, wilcoxon signed rank test with continuity correction indicated that there was a significant increase in PLE scores following d-amphetamine (V = 199, p = 0.00048), relative to placebo (presented in our previous report on funneling illusion [100]). As shown in Table 1, d-amphetamine significantly increased scores on the BPRS (V = 129, p = 0.001), MIS (V = 107.5, p = 0.039) and PAS (V = 127, p = 0.017), but not on the Launay-Slade (V = 75, p = 0.16) or SAPS (V = 124.5, p = 0.092) Out of the total of 20 participants, d-amphetamine increased schizotypy scores in 16 participants.

**Table 1:** Effects of d-amphetamine and caffeine on each Psychological scale.

Psychological Effects of Drugs				
Scales	Experiment 1 (M±SD)		Experiment 2 (M±SD)	
	D-amphetamine	Placebo	Caffeine	Placebo
**BPRS**	26.4±2.7**	23.7±1.1	25.1±2.0	24.8±1.3
**Launay**	3.1±2.7	2.6±2.1	0.9±1.7	1.2±1.6
**MIS**	4.6±4.0*	3.5±2.8	2.9±3.4	2.7±3.3
**PAS**	4.1±4.0*	2.5±2.8	2.5±3.1	2.0±2.0
**SAPS**	6.6±8.9	4.1±4.0	5.1±4.1	5.0± 3.6

** significant at p < 0.01

* significant at p < 0.05. Data are presented as Mean±SD.

#### PLE and working memory

Pearson’s correlation was used to assess how changes in PLE scores (d-amphetamine–placebo) relate to WM change scores (d-amphetamine–placebo). There was a significant negative correlation between d-amphetamine induced changes in PLE and changes in WM (maximum obtained) at the 8 s delay (r = -0.47, p = 0.03, n = 20), but not at 0 s (r = 0.28, p = 0.22, n = 20), 4 s (r = 0.09, p = 0.69, n = 20), or 6 s (r = 0.10, p = 0.67, n = 20).

To assess which span tasks influenced the association between the WM change and PLE change, we did separate analysis for each SWM and VWM at each delay condition. There was a significant negative correlation between changes in PLE and changes in SWM (r = -0.58, p = 0.006, n = 20), but not between changes in PLE and changes in VWM (r = -0.01, p = 0.96, n = 20) at the 8 s delay, indicating the correlation between WM and PLE was mainly influenced by SWM.

### Experiment 2: Caffeine

#### Caffeine effect on physiology

Analysis of variance with time and drug as within-subject factors and drug order as a between-subjects factor showed significant main effects of caffeine on systolic blood pressure (F[1, 19] = 27.9, p = 0.00004, ƞ^2^ = 0. 056) and diastolic blood pressure (F[1, 19] = 29.4, p = 0.00003, ƞ^2^ = 0. 055), but not on heart rate (F[1, 19] = 0.76, p = 0.39, ƞ^2^ = 0. 006) or temperature (F[1, 19] = 0.1, p = 0.88, ƞ^2^ = 9.3x10^-5^).

#### Caffeine effect on PLE

Wilcoxon signed rank tests with continuity correction revealed that there were no significant effects of caffeine on any of the PLE scales: BPRS (V = 87, p = 0.63), MIS (V = 70.5, p = 0.91), and PAS (V = 96, p = 0.35), Launay-Slade (V = 5.5, p = 0.17) and SAPS (V = 91, p = 0.82). Table 1 presents the effect of each drug on each PLE scale. As shown in Fig 4, unlike d-amphetamine, there was no significant increase in overall PLE scores following caffeine administration (V = 106, p = 0.98).

#### Caffeine effect on VWM and SWM

**Fig 5:**
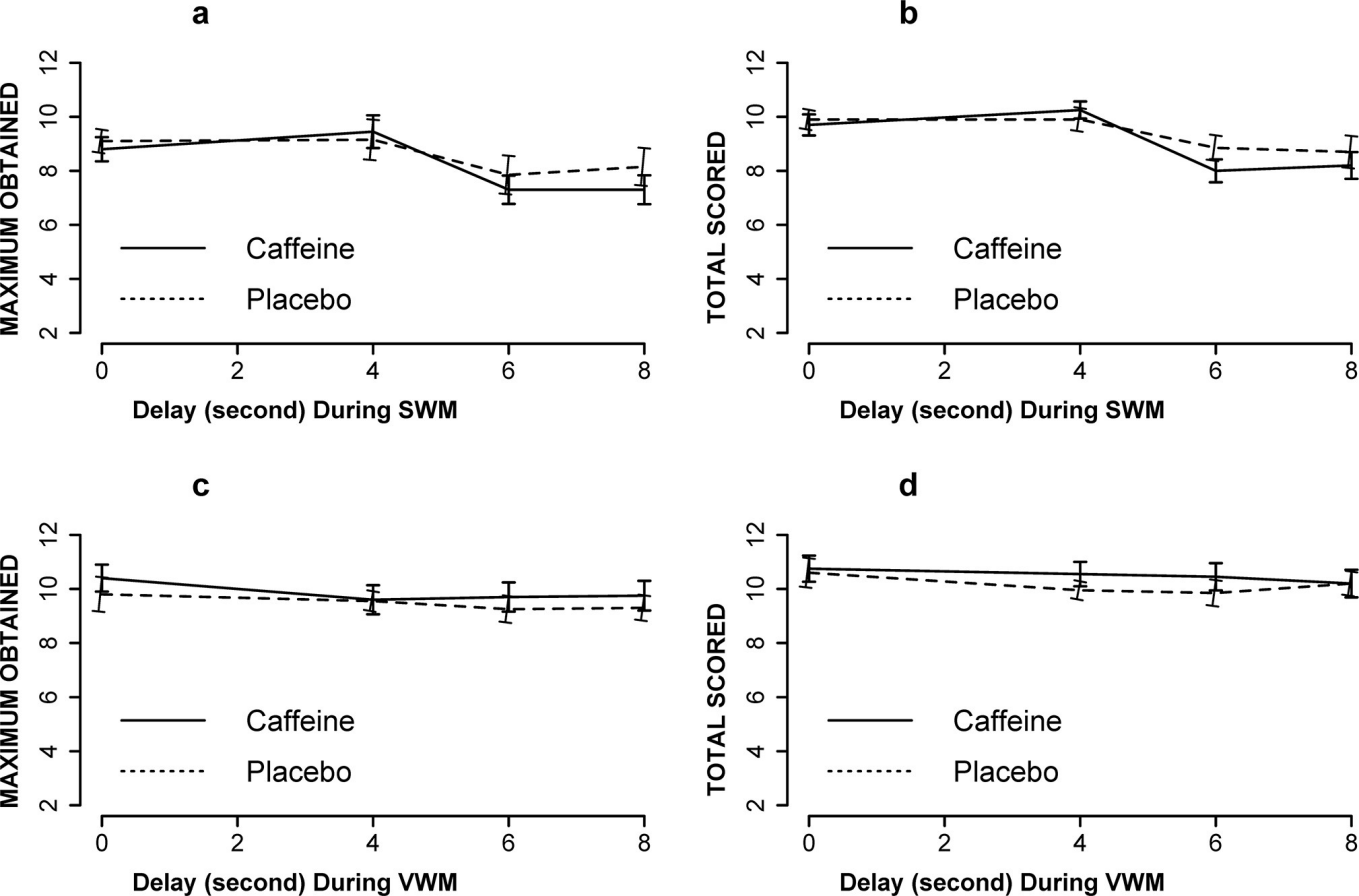
The effect of caffeine (200 mg, BID, PO) on SWM (a and b) and VWM (c and d) during forward tasks. Maximum obtained for SWM (a), total scored for SWM (b), maximum obtained for VWM (c), and total scored for VWM (d). There were no significant effects of caffeine on maximum obtained (a and c) and total scored (b and d) at each delay condition. (N = 20, for all).

ANOVA with delay and drug as within-subjects factors and drug order as a between-subjects factor did not indicate significant drug by delay interactions for SWM (maximum obtained F[3, 57] = 0.48, p = 0.69, ƞ^2^ = 0.007; and total scored F[3, 57] = 1.5, p = 0.22, ƞ^2^ = 0.04) or VWM (maximum obtained F[3, 57] = 0.23, p = 0.87, ƞ^2^ = 0.0022; and total scored F[3, 57] = 0.65, p = 0.58, ƞ^2^ = 0.0045). As shown in Fig 5, there were no significant effects of caffeine on SWM (maximum obtained F[1, 19] = 1.0, p = 0.3, ƞ^2^ = 0.0048); total scored F[1, 19] = 1.1, p = 0.30, ƞ^2^ = 0.006) and VWM (maximum obtained F[1, 19] = 1.4, p = 0.24, ƞ^2^ = 0.008; total scored F[1, 19] = 1.2, p = 0.28, ƞ^2^ = 0.007). Linear mixed effects model (with Satterthwaite’s method) failed to find interaction between session and drug on SWM (maximum obtained F[1, 72] = 1.0, p = 0.32); total scored F[1, 18] = 2.3, p = 0.15) and VWM (maximum obtained F[1, 18] = 0.03, p = 0.85; total scored F[1, 18] = 0.5, p = 0.48). There were also no effects of session on SWM (maximum obtained F[1, 72] = 1.8, p = 0.18); total scored F[1, 18] = 2.1, p = 0.17) and VWM (maximum obtained F[1, 18] = 3.1, p = 0.09; total scored F[1, 18] = 3.0, p = 0.09).

Although there were no main or interactional effects of caffeine on WM, SWM or VWM, there were significant effects of delay on SWM for the maximum obtained (F[3, 57] = 6.2, p = 0.0009, ƞ^2^ = 0.08) and total scored (F[3, 57] = 14.6, p = 3.4 x10^-07^, ƞ^2^ = 0.14). There were no delay effects on VWM for the maximum obtained (F[3, 57] = 1.22, p = 0.30, ƞ^2^ = 0.013) and total scored (F[3, 57] = 1.4 p = 0.25, ƞ^2^ = 0.01).

#### PLE and working memory

Although caffeine did not significantly influence PLE or WM, to compare the effects of caffeine and dexamphetamine we conducted the same correlation analysis. Changes in PLE scores (caffeine–placebo) were correlated with WM (maximum obtained) change scores (caffeine–placebo). There were no significant correlations between caffeine induced changes in PLE and changes in WM (r = -0.19, p = 0.40, n = 20), SWM (r = -0.11, p = 0.65, n = 20), VWM (r = -0.20, p = 0.38, n = 20). There was also no significant correlation between caffeine induced changes in PLE and changes in WM at each delay: the 0 s (r = -0.18, p = 0.44, n = 20), but not at 4 s (r = -0.25, p = 0.27, n = 20), 6 s (r = -0.23, p = 0.32, n = 20), and 8 s (r = 0.05, p = 0.84, n = 20).

## Discussion

The aim of the present two randomized studies was to investigate the effect of d-amphetamine (0.45 mg/kg, PO) and caffeine (200 mg, PO) on SWM and VWM performance in healthy subjects following delays in recall. The results showed that there are no main effects of d-amphetamine on the SST or the DST, relative to placebo. Likewise, caffeine did not affect performance on the SST or the DST, relative to placebo, which is consistent with previous reports on WM tests [38–41,50]. D-amphetamine, but not caffeine, increased PLE scores. The PLE effects of dexamphetamine in the present study is consistent with previous studies on psychosis-like symptoms or schizotypy ([see 75 for review, 76,77,101,102]). The results further revealed that there was a negative correlation between change in d-amphetamine–induced PLE and change in SWM, indicating that people with greater d-amphetamine-induced PLE demonstrated larger reductions in SWM performance. The lack of association between change in PLE and change in VWM might suggest that dexamphetamine, at the given doses, does not have direct or indirect influence on VWM in DST. Almost all of the studies that assessed the direct VWM effect of dexamphetamine failed to see any significant influence [5,13–15,22,31]. The selective effect of dexamphetamine on SWM might be because of dopamine-induced increases in spatial memory (or promotes spatial memory persistence) in the hippocampus [103–105] and PFC [67,106]. Importantly, the dopaminergic effect following dexamphetamine in the DLPFC might influenced SWM as previous studies showed that dopamine in the PFC have a crucial role for SWM performance in healthy individuals and people with schizophrenia ([for review, see 58,59–63]).

Although there are some studies that did not find effects of amphetamine on SWM, other studies did detect effects of amphetamine SWM ([6, See 12 for review, 16,17,19,22,107,108]). We show that d-amphetamine also indirectly influences SWM, based on PLE score. Previous studies found that, in addition to people with schizophrenia, subjects with high schizotypy have cognitive impairment ([109–112, for review, see 113–117]). Importantly, several studies reported that schizotypy is generally associated with impairments in WM ([see 118 for review, 119]) and other domains of cognition [120,121–125]. Schmidt-Hansen and Honey [114] and Rossi, Zammit [126] also found that reduced WM performance is associated with PLE in the general population, supporting the evidence that WM impairment may be a core feature in a subgroup of people with psychosis spectrum disorder. However, it has to be noted that some studies reported that there was no association between schizotypy and WM impairment on LN sequencing or DST [125,127,128]. Overall, the present indirect effect of d-amphetamine on SWM based on changes in PLE might be because of the change in the level of dopamine in the PFC as previous studies indicated.

### The effect of D-amphetamine and caffeine on WM

As there are no one generally accepted model to measure WM [55], various studies have used different tasks to measure SWM. For instance, the Cambridge Neuropsychological Test Automated Battery (CANTAB)-SWM test is commonly a non-verbalized task information that presents a number of boxes on screen and have been used to measure errors and response times, with better reliability [129]. The letter-number sequencing tests is also from the Wechsler Memory Scale version 3 that commonly used to measure auditory WM. The other subtest from the Wechsler Memory Scale version IV is n-back paradigm that use to measure SWM. The examinee during n-back tests judges whether each item (letter or picture) in a sequence is similar to the one that was presented n items ago ([for review, see 55]). The other prominent task to measure SWM performance (i.e., reaction times and accuracy) is The Sternberg Item Recognition Paradigm (SIRP) which is developed by Sternberg [130]. In SIRP, a series of items with different length are presented to the participants who are required, after certain maintenance or delay condition, to judge whether a test digit is contained in the memory sets [131]. Like the Spatial Delayed Response Task (SDRT) [132], one of the strength of SIRP is that it has delay condition that use to assess maintenance component of WM.

In the present study, we used DST and SST to measure VWM and SWM, respectively. The DST is a simple span tasks used to measure auditory WM. SST is comparatively more complex tasks [90], and it is not expected that the two tasks yield related results because they measure different domain/component of working memory [32]. Some researchers have suggested that digit tasks have lesser sensitivity compared to spatial tasks for measuring WM because forward digit tasks are simple tasks [133–135]. Both digit and spatial forward WM tasks areconsidered to measure “transient online storage”, while the backward conditions measure the “executive functioning” component of WM performance [135,136]. Relatedly, Frydecka, Eissa (99) and Haatveit, Sundet (133) described that the forward tasks measure the “load effect” (the load of information to be stored) and the backward tasks measure the “manipulation effect.” In addition to other dexamphetamine or caffeine studies on SWM and VWM measuring tasks, looking into other drugs sensitive to SST and DST are beneficial for comparison purpose. For instance, forward or backward DST and SST were applied to test the WM effects of other drugs such as THC [137–139] or nabilone [119,140]. Therefore, further studies on the sensitivity of dexamphetamine to DST and SST is valuable to have well stablished effects of dexamphetamine on verbal and spatial WM.

We failed to find a main effect of d-amphetamine on SWM, which is in agreement or comparable with previous studies on the SWM effect of d-amphetamine in various SWM task performances [5,16,21,26,27,29]. In contrast to the present finding, some other studies reported that d-amphetamine enhances SWM on various tasks such as SDRT, SIRP or n-back task [6,16,17,19,22,107,108]. Importantly, three studies that found the main or interactional effects of d-amphetamine on SWM used the SIRP task [19,22,107], which may indicate that SIRP is more sensitive to d-amphetamine. In addition, d-amphetamine enhanced SWM on n-back tasks [6,16]. However,other studies did not find effects of d-amphetamine on a similar task [5,16,26,27].

Although we used higher doses than previous studies that assessed the effect of d-amphetamine on SWM, we failed to find main effects of d-amphetamine on SST performance. In addition, we applied four delay conditions in order to examine the maintenance component of WM, but the results showed that d-amphetamine did not influence WM in the presence of delays. A previous study [108] that used a SDRT reported that d-amphetamine (0.25 mg/kg) enhances SWM only during a no delay condition (i.e., 0 ms), which might indicate that delay may not directly influence effects of d-amphetamine. These results together show that SST is not sensitive to detect direct effects of moderate to high doses of d-amphetamine on WM.

The present result also showed that d-amphetamine does not affect VWM on the DST under four delay conditions, which is in agreement with previous studies that used backward DST, forward DST or both to assess VWM [5,13–15,22,31]. One study that used backward and forward DST reported that d-amphetamine (10 and 20 mg) enhances VWM [18]. However, in addition to the DST, two other studies that used digit recall task and operation span task did not find effects of amphetamine on VWM. The lack of d-amphetamine effect on SST and DST may not be attributed to sample size or dose differences, as all of the previous studies except Wardle, Yang (16) that found main or interactional effects had similar or smaller sample sizes. Altogether, the results indicate that DST is not sensitive to d-amphetamine or d-amphetamine does not affect VWM with or without the presence of delay conditions.

With respect to caffeine, the present finding is consistent with previous reports on the acute effects of caffeine on SWM (n-back test) [38,39,50] and VWM domain [39–41]. Evidence generally indicate that caffeine has limited effects on tasks involving WM domain but it may influence WM through its effect on vigilance, attention or mood [36,48,51,52,141]. However, another study reported that high dose of caffeine (400 mg) but not low doses (100 and 200 mg) enhanced attention in habitual coffee consumers [84]. caffeine may negatively interfere with tasks that highly depend on WM ([for review, see 36]) and daily intake of caffeine containing beverages results in a worsening of WM performance on n-back tasks [42]. Based on reports from previous studies, it can be stated that caffeine directly facilitates lower-order cognitive performance such as reaction time or accuracy in attention tasks [36,37,43,52,53,142], indirectly influenceing WM through modulation of cortical and subcortical activations during WM processes [39,40,42,45,47] and/or simply counteractimg deteriorated cognitive performances under certain circumstances [143,144] Overall, the present results caffeine suggest that SST and DST may not be sensitive to caffeine, like d-amphetamine, or caffeine may not directly affect higher-order cognitive domains such as SWM and VWM performances.

### PLEs in response to d-amphetamine and caffeine

The present study showed that d-amphetamine (0.45 mg/kg, PO), but not caffeine (200 mg), increased PLE scores. Previous reports showed that the level of dopamine released after dexamphetamine administration correlates with PLE scores [75]. For instance, the released amount of dopamine in striatal and extra-striatal regions was found to correlate with PLE scores after d-amphetamine administration (0.43 mg/kg, PO) in sixty-three subjects [76]. The current study also found that a change in PLE is negatively associated with a change in SWM performance, which is comparable with previous findings in people with schizotypy [111,113,114,116], and people with schizophrenia [60,71,145–147].

In the present study, caffeine did not significantly affect PLE measures. However, we predicted that caffeine might slightly increase schizotypy scores as dopamine mediates some of the behavioural effects of adenosine antagonists [78,148]. Caffeine influences dopaminergic neurotransmission in different brain areas [34,80,149]. ([150,151, see 152 for review]). For example, caffeine modulate the release of dopamine through glutamate-dependent and glutamate-independent mechanisms [33], indicating that dopamine might play a role in the psychological (psychostimulant) effects of caffeine. Importantly, the striatal A2A-D2 receptor heteromer has been identified as a crucial pharmacological target for the psychostimulant effects of caffeine [34]. The psychostimulant effects of moderate doses of caffeine (200–300 mg) might also be through dopamine release in the thalamus [149]. In addition, other behavioural effects of moderate doses of caffeine in human (such as arousal) is likely through enhancing striatal dopamine D2/D3 receptor availability, rather than directly enhancing the release of dopamine [80]. We suggest that the lack of PLE effects in the present study indicate that the release of dopamine after 200 mg of caffeine might not be sufficient to affect dopamine activation enough to influence PLE.

There are several double-blind placebo-controlled studies in healthy participants that showed caffeine consumption increases subjective effects (such as arousal) [35,44,48,153,154]. Unlike our finding, Jones and Fernyhough [155] and Larrison, Briand [156] reported that caffeine consumption correlates with PLE scores on a single scale (on the Launay Slade Hallucination Scale and a positive symptoms scale of schizotypy, respectively). However, these two studies were not double-blind placebo-controlled experiments. Also, it is important to note that caffeine may increase psychotic experiences in high schizotypy participants [157,158] who may consume caffeine to alleviate their symptoms. However, neither Jones and Fernyhough [155] nor Larrison, Briand [156] address causation as they are correlational studies.

The present results also showed that caffeine increased physiological (blood pressure) measurements without having effects on WM, indicating that the drug was active during cognitive tasks. Previous studies reported that low to moderate doses of caffeine increase systolic and diastolic blood pressure ([159,160, see 161 for review]), although the effect of caffeine on heart rate (HR) is not consistent [159–162]. The adenosine inhibiting effect of caffeine is expected to increase the release of dopamine, noradrenaline and other neurotransmitters ([148,150, see 152,163 for review]). Thus, the increase in neurotransmitter release after a moderate dose of caffeine administration causes psychostimulant effects ([for review, see 148]) and peripheral vasoconstriction that increases blood pressure ([see 161 for review, 162]). To sum up, the results show that 200 mg caffeine changes physiological measures without affecting WM.

### Strength and limitations of the study

The main strength of the present study was its study design (placebo controlled, within subject studies). It investigated the potential effects of d-amphetamine and caffeine on SWM and VWM performance using SST and DST at different delay conditions. This study used d-amphetamine at a moderate-high dose (~33 mg), about two to three times higher than that used in most previous challenge studies. There are, however, limitations to be considered for future studies. First, the major limitation is the sample size; confidence would be increased with a larger sample size. Importantly, the sample size of female participants should be increased to identify sex-dependent effects as ovarian hormone may influence drug response [164–166]. Second, although each separate experiment was a within subject study, the comparison between d-amphetamine and caffeine was based on between subject studies. Third, we used only the forward recall direction of the span tests, which is easier than the backwards test. Fourth, it is also possible to question the time difference between the spatial task and the digit task, which might influence the effects observed in SWM and VWM. However, it should be noted that the drugs were active during the digit span and during other tests after the digit task. Notably, the physiological effect of d-amphetamine peaked at 110 and 210 min post-dose, which is also the approximate time for the digit and spatial span tests. Fifth, the double-blind may be compromised because of the participant’s ability to discriminate between active drug and placebo. The dosage (capsule) form used in this study might also impact the expectancy and placebo effect as the drink forms and coffee smells probably influence the effect and the environment. Sixth, it would be better to measure the plasma concentration of caffeine, d-amphetamine and metabolites [44,167] to compare individual differences in cognitive performances, although we did not take multiple measurements of each cognitive task. The other challenge that limit the interpretation is that d-amphetamine affects the release of other neurotransmitters such as noradrenaline [168] and, to a much lesser extent, 5-HT [169] in the PFC. Seventh, The effects may not be universal. Our study was limited to subpopulation of healthy volunteers, whose ages and education levels were very close, which make the participants a homogeneous population. Future amphetamine studies should conduct in elderly subjects as ageing is associated with cognitive decline [170], in a heterogeneous population and in amphetamine naïve subjects. Coffee metabolism is also altered with age in elderly people and they tolerate moderate amounts of caffeine (50–100 mg) [171]. Lastly, the current study did not verify the self-reports about recent substance use. However, a previous study in our lab that recruited participants from the general population found that self-reports about recent history of substance (cannabis) use are valid, with kappa = 0.91 [172].

### Conclusions

A moderate dose of d-amphetamine increased PLE on different scales. However, d-amphetamine does not directly affect performances on SST, but may impair SWM through its effects on PLEs that manifest during longer WM delays. In addition, moderate doses of caffeine do not affect performance on SST and DST. Overall, the present findings indicate that moderate dose of d-amphetamine and moderate doses of caffeine do not directly affect performances on DST or SST with and without delays.

## Supporting information

S1 File Consort 2010 checklist for dexamphetamine and caffeine studies. (DOC)

S2 File Dexamphetamine study participant information form. (PDF)

S3 File Caffeine study participant information form. (PDF)

S4 File Caffeine approval letter. (PDF)

S5 File Dexamphetamine and caffeine studies test description. (PDF)

S6 File Dexamphetamine study protocol. (PDF)

S7 File Dexamphetamine working memory data. (PDF)

S8 File Caffeine working memory data. (CSV)

S9 File Caffeine study protocol. (CSV)

## Abbreviations

DBP: Diastolic Blood Pressure
DLPFC: dorsolateral prefrontal cortex
PFC: prefrontal cortex
SWM: Spatial Working Memory
SBP: Systolic Blood Pressure
VWM: Verbal Working Memory
WM: Working Memory
