# Supplementary material for: The effects of caffeine and d-amphetamine on spatial span task in healthy participants
Source: PLoS One. 2023 Jul 13;18(7):e0287538. doi: 10.1371/journal.pone.0287538 (PMC10343048; doi:10.1371/journal.pone.0287538)
Supplement: S3 File — (PDF) [file pone.0287538.s003.pdf]

Chief Investigator:
Prof Mathew Martin-Iverson
Pharmacology, School of Biomedical Sciences, M510
The University of Western Australia
35 Stirling Highway, Crawley WA 6009
Tel: 6457-4569
Email:
mathew.martin-iverson@uwa.edu.au

Participant Information Form

Project title: Nabilone and caffeine effects on the perceptions of visually, auditory, tactile and multimodal illusions in healthy volunteers

Name of Researchers: Professors and Psychiatrists: Assoc. Prof. Jennifer Rodger, Prof. Joseph Lee, Dr. Rajan Iyyalol, Dr. Emily Hepple. Research Students: Jit Hui Mark Lim, Faiz Mohammed Kassim, Fui-Ling Voon, Benjamin Peters, Aakanksha Sharma.

Invitation:

“You are invited to participate in an honours project testing the effects of Nabilone or Caffeine on some perceptual illusions. You are asked to take part in this project because you expressed an interest in participating to one of the researchers.”

Aim of the Study (What is the project about?)

Our research project focuses on illusions known or thought to be experienced differently by people with schizophrenia (mental disorder characterised by delusions, hallucinations, paranoia and aberrant thought and language). These illusions include some auditory (Deutsch’s Phantoms Words), some tactile (Tactile Funnelling), and some that involve both, such as auditory and visual (the McGurk Effect), or visual and tactile (the Rubber and Projected Hand illusion).

These illusions are of interest because they reveal how the nervous system works, and from the basis of how we experience the world around us. One way to understand these mechanisms is to administer to healthy volunteers a drug, nabilone, that may change these functions, and to measure these changes. We are also interested in whether or not caffeine may alter these illusions.

We aim to determine if these drugs alter these illusions, similar to alterations in the illusions observed in those with certain psychiatric conditions, and similar to how another drug has been shown to alter them.

What does participation involve?

If you decide to participate, the study will take place over two testing sessions. Two testing sessions will both last approximately 7 hours. The Chief Investigator, or suitable proxy if CI is unavailable be present or nearby, and PhD research students will be present throughout the testing sessions, and one of the investigator psychiatrists will be on-call. The testing sessions will be about one week apart depending on your availability. Transport will be arranged for you to and from

the research lab. During each testing session you will swallow capsules containing placebo or nabilone (2 mg) in one experiment, OR in a different experiment, placebo or caffeine (400 mg). The total daily dose of nabilone will be similar to the daily dose to treat nausea and vomiting associated with cancer chemotherapy. The dose of caffeine is similar to two cups of strong espresso coffee. Caffeine capsules will be taken twice, in the morning and just before lunch (which we will provide for you), at least 3 hours apart. The order of the days in which you will get drug or placebo will be randomised and will not be revealed to you or the investigators until after both testing sessions have been completed (this 'double blind' can be broken if necessary).

Firstly, we will go over this information sheet with you to answer any question that you may have, and to ensure that you will understand what you will be doing. Then, you will take part in an interview with a psychiatrist to determine if you are eligible for the study. If you are eligible, basic information will be recorded about you such as age, height and weight. Then we will take three blood pressure and heart rate measurements, two body temperature measurements (with a probe that doesn't touch you, but measures the temperature of the blood in a vein in your forehead), and a saliva sample where we will ask you to spit at least 1 mL into a tube once each of the two test days (to get 2 mls). This saliva sample will be used to determine the type of gene you have for making the enzyme Catechol-O-methyltransferase, which varies amongst people and may be associated with differences in experiencing illusions. These samples will only be identified with a study identification code, not your name. You will be asked to fill in two drug scales (a stimulant scale and a marijuana scale). Physiological measures (body temperature, heart rate and blood pressure) will be taken and the stimulant and marijuana drug scales will be filled in 5 times per testing day.

Your employer may need to be informed of your participation (for example, should your workplace conduct drug testing). We can provide you with a signed letter describing your participation in this study, in this case.

You will then be given capsules to swallow with water, followed by a second capsule at a specific time later, both of which may contain either placebo or nabilone or caffeine. We will ask you to come for two day of tests, one week apart, because on one day you will have placebo and on the other day you will be give nabilone or caffeine. You will receive each for only one day (that is, placebo one day and nabilone the other, or, in another experiment, placebo one day and caffeine the other).

During most of the day, we will ask you to engage in some perceptual illusions, fill in some questionnaires, do some memory tests, and answer some questions about how you are feeling or thinking. Five times during the day, we will take blood pressure and body temperature and ask you to fill in questionnaires about how you are feeling. There will be a lunch break.

The second day will be the same as the first, except that you will not undergo a second interview with the psychiatrist. A description of the illusions and questionnaires that you will be asked to participate in can be provided to you if you wish.

After you have completed all of the tasks you will remain at the research centre until you feel well enough to go home, and the researcher is satisfied that your physical responses such as heart rate and blood pressure are normal and that you are not feeling the effects of the drug. In this time, it is advised that you do a quiet activity such as studying, reading or watching a movie.

Once an assessment indicates that it is safe for you to go home, transport will be arranged for you.

Voluntary Participation and Withdrawal from the Study

You may withdraw from the study at any time for any reason, and you are not required to give a reason. However, if you choose to withdraw after you have swallowed capsules containing nabilone or caffeine, you will have to remain at the research centre until the effects of the treatment have worn off, based on assessments by the researchers in charge or a psychiatrist to ensure it is safe for you to leave. We expect this to be 3 hours after you have swallowed the capsules. If it is deemed unsafe for you to leave, you will remain under the observation and be reassessed every 30 minutes.

There will be no consequences for your university studies or your medical care or for anything else to you, if you withdraw from the study, and if you do so without completing both days; tests, your data will be permanently destroyed, as will your saliva sample and the audio tapes. However, if you have completed some of the tests on both days, we would like to include your de-identified data in the study. However, you have the right to request that your data and saliva be destroyed, and we will comply with your request. Note, once we destroy the code sheet linking your name to the code that is stored separately from the de-identified data, your data will not be re-identifiable, and it will not be possible to remove your data from the study. That is, after we have completed the experiment, if you decide then to have your data withdrawn, it may not be possible to do so as it will be de-identified.

Your privacy

All information collected will be **coded**, such that your name is not associated with the information, and all the information will be treated as **strictly confidential**. These de-identified data will be stored securely and locked. De-identification data on computers will be password-protected. A sheet of paper that links your name to your code and that is kept in a locked cabinet in a locked room away from the data itself will be destroyed once the study is complete. There will be no record linking codes to specific individuals once this is done. De-identified results personal to you will not be given to any person other than the researchers involved in this study, unless required by law. Published data will be group data only, not individual data. De-identified raw individual data will be destroyed after 15 years. Your saliva sample will be identified only with a code that reveals no information about your identity. It will only be used to identify the type of Catechol-O-methyltransferase gene you have, and the saliva will be destroyed once that is done. The results of the genetic analysis will not have any information attached to it that could identify you, and it will only be used for the purposes of this study.

Possible Benefits

There are no direct benefits to you for participating, other than the value of the experience in learning about this kind of research afforded by your participation. We hope there will be benefits to the medical community and patients with psychotic disorders, to the level that we discover aspects of how brain networks are involved in psychotic illnesses, and the possible role cannabinoids have in this process. This may have implications for the use of medical marijuana, depending on the results.

Possible Risks and Risk Management Plan

Nabilone is a medicine that helps to reduce nausea and vomiting caused by many anti-cancer medicines. Nabilone is often used when other medicines have not alleviated the nausea or vomiting associated with chemotherapy. Nabilone is a synthetic (man-made) chemical known as a cannabinoid. It is not made from the cannabis plant, but is similar to some marijuana extracts and

can cause similar effects in humans. Like all medicines, Nabilone can cause side effects, although not everybody gets them.

Side effects that you may experience are:

- Feeling sleepy, relaxed, or “high”. A few patients have had hallucinations, felt confused, depressed, anxious or had other changes in their mood or mental state.
- A feeling of dizziness or spinning, especially when you stand up.
- Poor muscle co-ordination.
- Dry mouth, problems with your sight or concentration, difficulty sleeping, or headaches.
- Shaking, a faster heart beat than normal, low blood pressure, losing your appetite and stomach pains.

Caffeine also may have side-effects, although not everyone gets them. These include nervousness, irritability, sleeplessness and, occasionally, rapid heartbeat.

Any effects should wear-off in few hours. After you have completed all of the tasks you will remain at the research centre until you feel well enough to go home, and the researcher is satisfied that your physical responses such as heart rate and blood pressure are normal and that you are not feeling the effects of the drug. In this time, it is advised that you do a quiet activity such as studying, reading or watching a movie. If you feeling depressed and anxious at the end of the day, we can arrange to take you to student health services for counselling. If the student health services counselling is not available, the on-call psychiatrist can provide you with counselling. In the unlikely event of a serious adverse event, we will escort you to the Emergency Department of SCGH, or call an ambulance, as well as contacting the on-call psychiatrist.

Once an assessment indicates that it is safe for you to go home, transport will be arranged for you, as will transport to the testing facility.

You should not drive, operate machinery, or engage in any hazardous activity on the test days. Also, you should avoid alcohol, sedatives, hypnotics, or other psychoactive substances after we transport you home, as they may potentiate the central nervous system effects of nabilone.

Below is a list of over-the-counter medications that should not be used in the 48 hours before each testing session:

Antihistamines
Hayfever tablets (e.g. Telfast)
Codeine-containing medications
Cough syrups

A primary way we reduce the risks of adverse side-effects is through Inclusion and Exclusion Critieria. You may participate if you:

are between the ages of 18 and 59,

and are using contraceptives if female and sexually active and fertile. This is to exclude you from the experiment in case you have recently become pregnant but do not yet know of it.”

and ARE NOT:

pregnant or breast-feeding;
ingesting caffeine on the day of each testing session,
using current prescription medications other than oral contraceptives or acne medication
using over-the-counter medications in the 48 hours before each testing session

and do NOT have

Heart disease or severe blood vessel disease,
High blood pressure,
Hyperthyroidism,
Tics (muscle twitching usually in the face or shoulders)
Any degenerative disease of the nervous system,
Epilepsy, or other neurological disorders including head injury,
Tourette's syndrome or a family history of the disorder,
A psychiatric problem for which you are receiving treatment (schizophrenia, depression, anxiety, epilepsy, Parkinson's, etc)
A serious medical problem for which you are currently receiving treatment (cardiovascular disorders, respiratory disorders, ect),
Had or are currently receiving treatment for substance abuse,
A family history of schizophrenia in your first-degree relatives (parents, children or siblings),
A history of hypersensitivity to any cannabinoids (including cannabis),

Contacts

If you would like to participate or discuss any aspect of this study please feel free to contact by email

Mark 21470866@student.uwa.edu.au, Ben 21457829@student.uwa.edu.au,
Anu 21498272@student.uwa.edu.au, Fui Ling 22360949@student.uwa.edu.au
Faiz 22373904@student.uwa.edu.au or Mat mathew.martin-iverson@uwa.edu.au

Sincerely,

Professor Mathew Martin-Iverson

Chief Investigator

Approval to conduct this research has been provided by the University of Western Australia, in accordance with its ethics review and approval procedures. Any person considering participation in this research project, or agreeing to participate, may raise any questions or issues with the researchers

at any time. In addition, any person not satisfied with the response of researchers may raise ethics issues or concerns, and may make any complaints about this research project by contacting the Human Ethics office at UWA on (08) 6488 4703 or by emailing to humanethics@uwa.edu.au. All research participants are entitled to retain a copy of any Participant Information Form and/or Participant Consent Form relating to this research project.
