# Supplementary material for: The effects of caffeine and d-amphetamine on spatial span task in healthy participants
Source: PLoS One. 2023 Jul 13;18(7):e0287538. doi: 10.1371/journal.pone.0287538 (PMC10343048; doi:10.1371/journal.pone.0287538)
Supplement: S5 File — (PDF) [file pone.0287538.s005.pdf]

Dexamphetamine Effects on the Visual- and Auditory-Tactile Temporal and Spatial Stimulus Associability Windows

Description of tests and questionnaires

Spatial Span
The experimenter will tap a sequence of blocks on a grid. You will then tap, in the same order, those blocks. In some cases, you will be asked to wait a few seconds before copying the experimenter's taps. The number of blocks to tap will increase with each trial.

Phantom Word Illusion
A series of sounds will be played into both your ears through headphones. You will be asked to press a button whenever you hear a word other than two specific words, and to say out load what word you hear, which will be recorded for later analysis and then destroyed after coding and auditing of the coding.

Visual Induced Flash Illusion
A series of lights will flash briefly on a video monitor, some at a fixation point, and others further away. You will be asked to press a button representing the number of flashes you observed at the fixation point.

Rubber Hand Illusion
In this test, you will view a rubber right hand while your own hand is hidden from your view. Both Rubber hand and your hand are stimulated simultaneously with a light brushstroke from a paintbrush. Before and after the brushstroked, you will fill in a questionnaire about how you felt about your hand. This task will be repeated a few more times with different the rubber hand at different distances from your own hand. .

Digital span test
You will see a sequence of digits on a video monitor, and will be asked to repeat them in order. In some cases, you will be asked to wait a few seconds before repeating the digits. The number of digits to recall will increase with each trial.

Marble Hand Illusion
In this task, you are blindfolded and your hand is tapped with a hammer while you focus on the sound of a hammer tapping a hand through a headset. You will fill out a questionnaire before and after each set of taps.

Tactile Funneling Illusion
You will see a sequence of digits on a video monitor, and will be asked to repeat them in order. In some cases, you will be asked to wait a few seconds before repeating the digits. The number of digits to recall will increase with each trial.

McGurk Effect
You will videos on a video monitor of someone saying a syllable, and hearing a syllable through headphones, and will be asked to repeat the sounds you hear out loud. These will be recorded and coded offline. After auditing the data, the audio recordings will be erased.

Questionnaires and Interviews
Over the course of the experiment, you will be asked to fill in questionnaires or answer questions. Two will be brief interviews (BPRS and SAPS) about how you feel on that day. The MIS will ask true/false questions about some beliefs and experiences you may or may not have. The PLE and PAS questionnaires will ask you about any out of the ordinary perceptions you may have.
We will ask you to fill in the amphetamine scale 5 times during the day, to determine if there are any changes in amphetamine-related effects that this scale assesses. We will also ask you to fill in a brief questionnaire on anxiety at the same times. We will also ask you to spit into a vial 5 times during the day. The saliva samples will be used to measure the levels of dexamphetamine, and we will measure your blood pressure, heart rate and body temperature (the latter noninvasively) at the same times that you fill in the amphetamine scale.
