# Supplementary material for: The effects of caffeine and d-amphetamine on spatial span task in healthy participants
Source: PLoS One. 2023 Jul 13;18(7):e0287538. doi: 10.1371/journal.pone.0287538 (PMC10343048; doi:10.1371/journal.pone.0287538)
Supplement: S6 File — (PDF) [file pone.0287538.s006.pdf]

Human Ethics Office
Office of Research Enterprise, MBDP: M459
The University of Western Australia
35 Stirling Highway
Crawley WA 6009
Phone: +61 8 6488 4703
Fax: +61 8 6488 8775
Email: humanethics@uwa.edu.au
research.uwa.edu.au/staff/human-research

Amendment Request - Human Research Ethics

Use this form to request approval for an amendment to an existing human ethics approval provided by The University of Western Australia. If your original approval is from another HREC, but has been *recognized* by UWA, you need to apply to the original HREC for amendments.

Note: *Significant amendments to a project may require submission of a new Human Research Ethics Application.*

Submit completed form to Human Ethics office: humanethics@uwa.edu.au

**UWA reference no.**	RA/4/1/8056

**1.Project title:**	**Dexamphetamine effects on the Visual- and auditory-Tactile Temporal and spatial stimulus associability windows**

**1.Chief Investigator, or Supervisor in case of student research:**			
**Name**	Professor Mathew Martin-Iverson<br>*Title, given name, and family name*		
**Telephone**	6457 4569	**Email:**	mathew.martin-iverson@uwa.edu.au

Changes to Investigator List:

Additional researchers / co-investigators, including students and non-UWA investigators

Researcher	Title, given name, and family name	School, centre, institution	Email	Co-inv, student,
1	Dr Emily Hepple	North Metropolitan Health Services: Mental Health	Emily.Hepple@health.wa.gov.au	Co-investigator
2	Dr Salam Hussain	Psychiatry, School of Medicine	salam.hussain@uwa.edu.au	Co-investigator
3	Mr Jit Hui Mark Lim	Pharmacology, School of Biomedical Sciences	21470866@student.uwa.edu.au	student
4	Mr Sean Loffman	Pharmacology, School of Biomedical Sciences	21323839@student.uwa.edu.au	student
5	Ms Sophie Slawic	Pharmacology, School of Biomedical Sciences & Psychology, University of Bremen	sslawik@uni-breme.de	student
6	Ms Katharina Gaus	Pharmacology, School of Biomedical Sciences & Psychology, University of Bremen & Psychology, University of Bremen	ka_ga@uni-bremen.de	student

Researchers no longer work on this project, including students and non-UWA investigators

Researcher	Title, given name, and family name	Date no longer associated with project		
1	Jason Lei	26 April	2017	student

List all changes to the project:	
	1. Rewritten the first paragraph description of the project in attachment 5A (participant information form) and changed the list of investigators and the affiliation of the Chief Investigator on both attachment 5A (PIF) and on the participant consent form (Attachment 2).<br>2. Rewritten descriptions of tests in attachment 5C to be less descriptive of our intended experimental outcomes but still be descriptive enough for the participant to know what it is.<br>3. Dr Emily Hepple, Dr Salam Hussain, Mr Jit Hui Mark Lim, Mr Sean Loffman, Ms Sophie Slawic and Ms Katharina Gaus added to the list of investigators. Mr Jason Lei removed from project, as he has withdrawn from his PhD candidacy.<br>4. Rewritten and updated the PIF to provide a better initial description and include the additions and changes to the experiment.<br>5. Added in the Marble Hand Illusion. The marble hand illusion involves tapping the participants' hand with a hammer while they listen to the sound of a soft 'fleshy' tap that gradually changes to a harder tap sound as though the hammer is tapping marble and another condition in which the sound doesn't change from the sound of flesh being tapped. Last year's result indicate that the temporal binding window in this illusion may be altered by dexamphetamine.<br>6. Added in the Visual Induced Flash Illusion.<br>7. Removed the peak interval procedure.<br>8. Added in the digital and spatial span tests from the WSM-III. The spatial test involves the participant reproducing the touches to specific blocks in an array of identical blocks made by the experimenter. The digital span is requires verbal repetition of a list of heard digits. With each success, the length of taps or number of digits needed to be reproduced is increased. In addition, 3 different delays between the presentation and the response are interposed.<br>9. Changed the anxiety questionnaire from the STAI to a shorter state anxiety questionnaire by Marteau and Bekker (Attachment 15).<br>10. Changed the Projected Hand Illusion to the rubber hand illusion, removed the delay component (for which we already have sufficient sample for both the rubber hand (published) and projected hand illusion (in preparation) and changed the distance variables to 15, 30, 45 and 60 cm hand offset from the image.<br><br>Provide details of the changes you propose.

Reasons for changes:	Summary of changes:<br>The general protocol remains the same especially in terms of those with ethical implications, as does the hypotheses being tested (that dexamphetamine increases temporal and spatial stimulus binding, thereby increasing the subjective experience of certain perceptual illusions dependent on such binding). Minor changes in wording of descriptions to participants have been included to reduce possible biasing responses in a particular direction. Some personnel have been added and removed to account for changes in students and to increase the number of psychiatrists (two more added) on the protocol because of high clinical loads imposed on them making regular attendance for screening intervals difficult. Some of the specific tasks have been changed due to recent findings from our previous dexamphetamine protocol that we have established in 2016, putting in ones on which we found possible significant drug effects on, dropping some that had no indication of drug effects, and adding two memory tasks that may provide a mechanism for the increased temporal and spatial binding in the perceptual illusions that reflects an additional hypothesis arising from the data collected in 2016 from a previous dexamphetamine protocol RA/4/1/7557, now complete. The additional hypothesis is that the temporal and spatial binding increases are due to increased spatial working memory. The changes are minor in regards to no changes in the ethical consequences for the participants, including burden of testing, and involving changes in personnel and refinement of some of the behavioural tasks in order to keep the same actual time by participants but maximise the quality of the information gained thereby keeping to the scientific aims that have been approved but enhancing the scientific impact.<br>Specific changes are addressed below:<br>**1.** Wording was changed to make the description of expected experience from the experiment less specific to reduce biasing responses. Changes in University structure due to the restructure, and changes in personnel have been made on the PIF and the PCF.<br>**2.** As in 1 above.<br>**3.** We added two more psychiatrists due to limited availability of specific psychiatrists due to their heavy clinical workloads. We also added this years' current honours students and masters students, and removed Jason Lei who has withdrawn from PhD studies.<br>**4.** Updated schedule for the participant.<br>**5.** Our previous research found that dexamphetamine is effective in influencing a number of illusions involving somatosensation and/or spatial separation of the illusion inducing stimuli. The Marble Hand Illusion involved a somatosensory and auditory component, while the rubber hand and Projected Hand Illusions is primarily somatosensory and visual.<br>**6.** We have found preliminary evidence of dexamphetamine-induced increased illusory effects in the visual modality for illusions that contain a spatial component (the visual induced flash illusion) but without a somatosensory component. Similar effects were not observed in visually similar visual flash without a spatial component. The sample size for the finding of significant effects in the visual flash illusion needs to be increased to establish confidence in the preliminary finding.<br>**7.** The time interval estimation task was removed because no indication of an effect was observed in a previous experiment, and due to a shortage of time with the additional memory tests (see 8).<br><br>Provide reasons why the changes are necessary.

Reasons for changes:	
	**8.** The spatial span test was added because of the finding that all illusions affected by dexamphetamine involved spatially distinct stimuli but not similar illusions without a spatial component. We hypothesise that this may be due to dopamine-induced increases spatial memory, as primate studies have shown that prefrontal dopamine has a direct effect on spatial memory (Sawaguchi T and Goldman-Rakic PS (1991) D1 dopamine receptors in prefrontal cortex: Involvement in working memory. Science 251:947-950; Williams GV and Goldman-Rakic PS (1995) Modulation of memory fields by dopamine D1 receptors in prefrontal cortex. Nature 376:572-575), and that patients with schizophrenia exhibit spatial working memory deficits (Keefe RS, Roitman SE, Harvey PD, Blum CS, DuPre RL, Prieto DM, Davidson M and Davis KL (1995) A pen-and-paper human analogue of a monkey prefrontal cortex activation task: Spatial working memory in patients with schizophrenia. Schizophr Res 17:25-33), that are also observed in relatives of schizophrenics, indicating that it may not be induced by medications (Park S, Holzman PS and Goldman-Rakic PS (1995) Spatial working memory deficits in the relatives of schizophrenic patients. Arch Gen Psychiatry 52:821-828), and these deficits are improved by antipsychotic (dopamine receptor antagonists) that correlates with an improvement in symptoms (Park S, Püschel J, Sauter BH, Rentsch M and Hell D (1999) Spatial working memory deficits and clinical symptoms in schizophrenia: A 4-month follow-up study. Biol Psychiatry 46:392-400). These data have led to an additional hypothesis by us that the effects of dexamphetamine on the experience of the illusions is due to an increase in strength and duration of spatial working memory, and that the increased temporal and spatial stimuli binding is due to this increased duration of spatial working memory. The digit span task is a control condition that involves nonspatial working memory and is included as a control task.<br>**9.** This is a shortened version of the STAI Y1 (anxiety state inventory). Scores obtained in this short form have been found to be very well correlated with the full form. The advantage of this change is shorter time taken to fill out the questionnaire multiple times throughout the day. Additionally, it will be less of a burden for the participants to fill in a 6 item questionnaire several times over the day compared to a 20 item questionnaire.<br>**10.** Following from the increased effects of spatial perception due to dexamphetamine, we would like to investigate the rubber hand illusion at a range of distances to determine the effect of dexamphetamine on the spatial binding window for this illusion. We are switching to the rubber hand illusion as there has been previous work with the rubber hand illusion indicating that the 30 cm is the usual spatial limit for the illusion to occur normally (Lloyd DM (2007) Spatial limits on referred touch to an alien limb may reflect boundaries of visuo-tactile peripersonal space surrounding the hand. Brain Cogn 64:104-109), whereas there is no previous research on the spatial constraints for the projected hand illusion. There is also more general data available on the rubber hand illusion. Previous research in our lab with the projected hand illusion indicated no dexamphetamine effects on the agency measure, which is the measure on which the projected hand illusion differs from the rubber hand illusion. Previously, we have used the rubber hand illusion at 15 cm visual offset between hand and screen and varied the timing between the real hand strokes and the rubber hand strokes. Now we will investigate distances of the rubber hand form 15, 30, 45 cm, or 60 ms from the participants' real hands with simultaneous stroking, as used by Lloyd (2007) to determine if dexamphetamine increases the spatial binding limits for the rubber hand illusion, as the temporal binding limits have been shown to be increased previously (Albrecht MA, Martin-Iverson MT, Price G, Lee J, Iyyalol R and Waters F (2011) Dexamphetamine effects on separate constructs in the rubber hand illusion test. Psychopharmacology (Berl) 217:39-50.)

**Impact on project documents:**	**1.** Attachment 5A (PIF) introductory paragraph rewritten. Personnel and responsible university division/school have been changed on PIF (Attachment 5A) and PCF (Attachment 2)<br>**2.** Updated attachment 5C (test description) to include a description of the Marble Hand Illusion, visual induced flash illusion and working memory tests. Changed description of the Projected Hand Illusion to the rubber hand illusion.<br>3. Updated attachment 5B (test schedule) to reflect the experiment changes and additions<br>4. Changed the State-Trait Anxiety Inventory (attachment 13) to the shortened version by Marteau and Bekker.<br>What changes will be required to existing project documents (e.g. information and consent forms, etc.) Attach copies of modified documents to this Amendment application.

**Potential impacts on participants:**	**1. There should be no impact on the participants during the testing in terms of effort or time spent testing, or on risk.**<br>**2. Participants will have less chance of bias to respond to tasks in specific ways from expectations from the PIF.**<br>Indicate any potential inconveniences, discomforts, harms or risks to participants.

**Actions taken to manage risk:**	Risk is managed better by having a wider pool of experienced clinicians to assess risk before admitting prospective participants to the study.<br>Indicate any additional actions or support that you need to provide as a result of the changes.

**Expected date of implementation:**	**As soon as formal approval from the HREO is granted.**<br>You cannot implement the requested changes before you receive formal approval from the HREO.

**Potential legislative requirements:**	None different<br>Please list if appropriate, e.g. do requested changes affect Privacy Act or Working With Children requirements?

Please attach the following:

1. New or modified documents such as the Participant Information Form (PIF) or Participant Consent Form (PCF).
2. Electronic copy of any other relevant documentation such as letter from governing authority or email correspondences.

The following paragraph must be included in all **Participant Information Form (PIF) and Participant Consent Form (PCF)**:

"Approval to conduct this research has been provided by the University of Western Australia, in accordance with its ethics review and approval procedures. Any person considering participation in this research project, or agreeing to participate, may raise any questions or issues with the researchers at any time.

In addition, any person not satisfied with the response of researchers may raise ethics issues or concerns, and may make any complaints about this research project by contacting the Human Ethics Office at the University of Western Australia on (08) 6488 3703 or by emailing to humanethics@uwa.edu.au

All research participants are entitled to retain a copy of any Participant Information Form and/or Participant Consent Form relating to this research project."

Certification / signature

**Chief Investigator or Supervisor of Higher Degree Research Student:**	**Declaration by the Chief Investigator**<br>• I declare that the information in this form is accurate and complete.<br><br>**Signed:**<br><br>**Name:** Mathew Martin-Iverson **Date:** 22.06.2017

UWA policy deems this document as signed if you send it attached to an email **from your UWA email address**. Alternatively, you can sign the signature page, scan that page and send it with the other pages and application documents.
