# Supplementary material for: The effects of caffeine and d-amphetamine on spatial span task in healthy participants
Source: PLoS One. 2023 Jul 13;18(7):e0287538. doi: 10.1371/journal.pone.0287538 (PMC10343048; doi:10.1371/journal.pone.0287538)
Supplement: S7 File — (PDF) [file pone.0287538.s007.pdf]

Human Ethics Office
Research Services, MBDP: M459
The University of Western Australia
35 Stirling Highway
Crawley WA 6009
Phone: +61 8 6488 4703
Fax: +61 8 6488 8775
Email: humanethics@uwa.edu.au
research.uwa.edu.au/staff/human-research

Application for Human Research Ethics Review

(Please use the latest version of this form every time you submit an application)

Use this form to apply for ethical review of research involving people to be carried out at The University of Western Australia. Ethical review of research is a necessary pre-requisite for research involving humans at the University. This includes even research that studies data about people and observation of people.

Submit completed form to Human Ethics office: humanethics@uwa.edu.au

Email or phone the office for assistance with this form – contact details are in the letterhead above.

**Do you seek Exemption from Review?**	**No**<br><br>**If Yes, stop here**. Please use the form **Application for Exemption from Ethics Review**, available at the Human Research Ethics website (click here).

**Have you received ethics approval from another Ethics Review Committee?**	No<br><br>• If you have Ethics approval from another Australian HREC that is registered with the NHMRC , you could apply for a UWA approval through recognition, please use the form **Application for Recognition of another ethics approval**, available at the Human Research Ethics website (click here); If you still plan to seek UWA approval for a project already approved by another Australian HREC please contact the Human Ethics office;<br>• If another HREC is reviewing your project and you wish to submit the same project to UWA HREC, please **complete this form and provide the reason** you are applying to two HRECs;<br>• If you have ethics approval from an overseas HREC and the application for UWA approval is for a new research student project, please **complete this form and provide the existing approval as supporting documents.**<br>• If you have ethics approval from an overseas HREC and the application for UWA approval is to satisfy a requirement to release grant funds, please **contact Human Ethics office before lodge - in an application.**

Continue below if you seek UWA review of your human research ethics project.

**1. Project title:**	Nabilone and caffeine effects on the perceptions of visually, auditory, tactile and multimodal illusions in healthy volunteers

**2. Chief Investigator, or Supervisor in case of student research:**			
**Name**	Professor Mathew Martin-Iverson		
**School, Centre or Faculty**	Pharmacology, School of Biomedical Sciences	**Staff Id #:**	00029004
**Telephone**	+61 8 9347 6443	**Email:**	mathew.martin-iverson@uwa.edu.au

Additional researchers / co-investigators, including students and non-UWA investigators			
**Researcher**	**Title, given name, and family name**	**School, centre, institution**	**Email**
**2**	Assoc. Prof. Jennifer Rodger	Neuroscience, School of Human Sciences, UWA	jennifer.rodger@uwa.edu.au
**3**	Prof. Joseph Lee	School of Psychiatry UWA	joseph.lee@uwa.edu.au
**4**	Dr. Rajan Iyyalol	North Metropolitan Health Service, Mental Health	rajan.Iyyalol@health.wa.gov.au
**5**	Dr. Emily Hepple	North Metropolitan Health Service, Mental Health	emily.hepple@health.wa.gov.au
**6**	Jit Hui Mark Lim	School of Biomedical Sciences, Pharmacology, UWA	21470866@student.uwa.edu.au
**7**	Faiz Mohammed Kassim	School of Biomedical Sciences, Pharmacology, UWA	faizmohammed.kassim@research.uwa.edu.au
**8**	Fui-Ling Voon	School of Biomedical Sciences, Pharmacology, UWA	22360949@student.uwa.edu.au
**9**	Benjamin Peters	Neuroscience, School of Human Sciences, UWA	21457829@student.uwa.edu.au
**10**	Aakanksha Sharma	Neuroscience, School of Human Sciences, UWA	21498272@student.uwa.edu.au

[Insert additional rows for additional investigators] emails for UWA staff & students are sent to the UWA email account

Hyperlinked cross references to the *National Statement on Ethical Conduct in Human Research* are abbreviated below as NS §section.chapter. If you have any doubt about the meaning or purpose of a question, please use those hyperlinked references to read the definitive explanation.

**3. Is this a Student Project?**	Honours ☑ Masters ☐ PhD ☑ No ☐ Other ☐	
	• *Provide the approved research proposal*<br>• *Provide Student name & number*	

4. **Team Expertise**	**Professor Mathew Martin-Iverson**<br>Principle supervisor. Psychopharmacologist with expertise in pre-clinical and clinical research of psychiatric disorders and drug effects. Has published 96 peer-reviewed papers, 7 book chapters, 2 books as editor, 6 State Clinical Guidelines, 42 published conference abstracts, mainly on dopamine-related research on schizophrenia, drug addiction and Parkinson's disease. Primary advisor for research design, data analysis, and paper writing.<br><br>**Assc. Prof. Jennifer Rodger**<br>Co-supervisor. Neuroscientist with expertise in Biochemistry and Molecular Neuroscience. Has published 93 peer-reviewed papers and a teaching publication. Key research is in the topographical organization of the brain, structure-function relationships and brain plasticity in development and repair.<br><br>**Prof. Joseph Lee, Dr. Rajan Iyyalol and Dr. Emily Hepple** are psychiatrists who were the medical personnel on the previous dexamphetamine studies, and are continuing to be co-investigators. Prof. Lee has a great deal of experience in drug clinical trials and other research in psychiatry, as well as experience in previous dexamphetamine trials in healthy volunteers in our lab. Prof. Joseph Lee will be the primary holder of the nabilone authority, and Prof. Martin-Iverson will also hold a Schedule 8 Permit for nabilone.<br><br>**Mr. Lim Jit Hui Mark, 21470866, PhD student**<br>Has previous experience in basic human drug research and pharmacology techniques from his undergraduate degree and honours project that involved similar testing with dexamphetamine in humans. PhD proposal will be forwarded when completed.<br><br>**Ms. Fui-Ling Voon, 22360949, PhD student**<br>Has previous experience in research and pharmacological techniques from her undergraduate and Master's degrees. PhD proposal will be forwarded when completed.<br><br>**Mr. Faiz Mohammed Kassim, 22373904, PhD student**<br>Has previous experience in pharmacology research from his Master's degree, and previous employment in an academic and research center. Has published 5 peer-reviewed papers and 2 international conference abstract papers and a presentation, on neuropharmacology. PhD proposal will be forwarded when completed.<br><br>**Mr. Benjamin Peters, 21457829, honours student**<br>Has previous experience in basic research, psychological science and neuroscience techniques from his undergraduate degree. Has approved project for an honours in Neuroscience, attached.<br><br>**Ms. Aakanksha Sharma, 21498272, honours student**. Has previous experience in basic research and neuroscience techniques from her undergraduate degree. Has approved project for an honours in Neuroscience, attached.<br><br>NS §3.3.5, *Explain how the research team has sufficient skills and experience to conduct the proposed research.*

5. **Expected project end?**	End date:	31/12/2022

**6. How is the research funded?**	School of Biomedical Sciences, Pharmacology postgraduate student research budget.<br><br>Please provide project application title for funding if different from this application.<br>Funder, Scheme, reference number (as applicable). Please provide UWA grant reference number if applicable: RA/1/####/###<br>Is funding sought that is not yet approved? Respond to the potential conflict of interest question at the end of this form.<br>Funded by business unit 00885, PG # 10400148

**7. Aims of this project. (Layman's terms)**	We, and others, have shown that people with schizophrenia, and healthy people given a sub-psychotic dose of dexamphetamine show greater susceptibility to a number of perceptual illusions when the stimuli that produce the illusions are separated in time and/or space. This separation of stimuli in time and/or space would be too much which ends up in the production of illusions in healthy controls (or after placebo in the dexamphetamine case). For example, in the rubber hand illusion, a rubber hand becomes perceived as one's own hand. This occurs when one's own hand cannot be seen but is brushed simultaneously as an observed brushing of the rubber hand. This only occurs if the two "hands" are brushed within 200 ms of each other and the rubber hand is within 30 cm of one's own hand. However, patients with schizophrenia and healthy volunteers given a moderate dose of dexamphetamine continue to experience this illusion, and the others described below, when the stimuli are further apart in time and space than would normally work (for hand illusions, even at 500 ms separation, and 45 cm distance).We have interpreted these observations to indicate that psychosis is related to an increase in binding windows, the limits of differences in time (temporal binding windows) and space (spatial binding windows) that stimuli have to be within to be perceived as a whole, single object. Our research with dexamphetamine points to dopamine as the neurotransmitter that increases the limits of the binding windows, and likely is related to dopamine's roles in attention and spatial working memory. There is current interest in the relationship between cannabis use and schizophrenia. Therefore, we plan to test the effects of a synthetic cannabis-like drug (nabilone) on measures that we found dexamphetamine significantly influenced. If nabilone increases binding windows, those results support that cannabis is causally related to psychotic symptoms. However, if nabilone decreases the limits of binding windows, then it may have antipsychotic effects. Nabilone is similar to Δ^9-tetrahydrocannabinol (Δ^9-THC) in structure and function, and has been a schedule 8 drug available for medical use as a treatment for chemotherapy-induced nausea in Australia for more than 20 years. It has a long history of medical use so the risks associated with the clinical dose (2 mg, twice a day), which are surprisingly low for a drug, are well-characterised and generally absent or minor. It has been used in research previously with at least 10 human research reports, including in research on illusions. One possible issue is that our placebo is not an active placebo. It may be that effects on illusions are due to people detecting subjective effects, and then change their reporting of illusion experience due to that difference between placebo. Therefore, we will conduct another experiment comparing caffeine (200 mg, twice a day) with placebo. If caffeine has no significant effects, we can use it in future experiments as an active placebo.

**8. Research design (include analysis & procedures if any)**	We will follow a double-blind, placebo-controlled, balanced cross-over design. We have run this drug protocol for seven years with dexamphetamine (but with different behavioural/ electro-physiological measures for 4 years and with similar measures for 3 years) with no adverse events. Essentially, half of the participants receive placebo on the first day, and nabilone (2 mg, taken orally, twice a day – the average clinical dose) on the second day, approximately 1 week later, while the other half of the participants receive nabilone first and placebo second. This follows the dexamphetamine experiment protocol, such that the results will be comparable. The exact same design will be used in a separate experiment, replacing nabilone with caffeine (200 mg/kg, taken orally, twice a day), to determine if caffeine may be suitable as an active placebo. If caffeine is suitable, then we will repeat the experiment with nabilone, only using caffeine as the placebo control (active placebo). Therefore, each participant is his/her own control. Basic demographic information (age, years of education, sex, height and weight, Edinburgh Inventory for Handedness) and the rating scales: Brief Psychiatric Rating Scale (BPRS) Scale for the Assessment of Positive and Negative Symptoms (SAPNS)), Revised Slade-Launay Hallucinations Scale (RSLHC). Magical Ideation Scale (MIS), Perceptual Aberrations Scale (PAS), State-trait anxiety inventory (STAI) will be conducted once each day. Blood pressure, body temperature and the Amphetamine Mood Questionnaire (AMQ: McKetin et al., 1999), Marijuana Mood Questionnaire (MMQ: Lile et al., 2009) and Marteau-Bekker self-evaluation questionnaire (Marteau and Bekker, 1992), assessing positive symptomology and amphetamine-withdrawal symptoms, will be measured 5 times each day to follow time-course of drug effects.<br><br>Illusions tested have all shown effects of dexamphetamine and include: **Projected hand illusion (PHI)** {Graham-Schmidt et al., 2016, Conscious Cogn, 45, 9-23} and the **rubber hand illusion (RHI)** {Albrecht et al., 2011, Psychopharmacology (Berl), 217, 39-50}, both of which will involve temporal separation by 0 and 500 ms, and spatial separation of 15, 30, 45 and 60 cm. Responses are changes in established questionnaires assessing illusion experience (post-illusion – pre-illusion responses). **Phantom Words Illusion (PWI)**, two different auditory stimuli ("Harvey" and "High-Low") will be presented to the participant via headphones at approximately 100 dB. Each word is one octave apart, and will alternate between 400 and 800 Hz between ears. Delays between 220, 440, 660, 880 and 1100 ms will be included between the left and right ears. The participants speak out loud sounds that they hear other than Harvey and High-Low, and the number of words heard is the major dependent measure. **Visually-Induced Flash Illusion (VIFI)** in which participants presented with two inducer flashes and a single target white Gaussian blobs flash on a PC screen. Flashes are white Gaussian blobs for 16ms against a black background and are presented for 16 ms against a black background. The second inducer flash will vary in ISI (0, 17, 25, 33, 50, 100, 150, 200 ms) and vertical position (3°, 5°, 7°, 9° eccentrically below the fixation flash) across trials, with all conditions randomly presented. The response is the number of flashes in the target location the participant reports seeing. **Tactile Funnelling Illusion (TFI)** in which a modified compass instrument will be used to apply two points of tactile stimulus either synchronously or asynchronously to a participant's forearm. Distance and time between compass points will vary throughout the experiment with time between 0-750 ms, and distance 1-5 cm. **McGurk Effect (ME)** in which an audio-visual presentation will be presented of the face of actors (1 female, 1 male) pronouncing a syllable on a screen, while another syllable is heard through headphones at approximately 100 dB. The visual stimuli and the auditory stimuli are separated in time for 0, 400, 500, 600, 700, 800, 900 and 1000 ms. The participants state out loud what is heard, and their response is coded as visual (heard the syllable mouthed), auditory (heard that sound played in the headphones) or chimeric, if neither of those two. Memory tests are the **Digital Span (DS)** working memory test, with delays between hearing the digits and recalling them will 0, 4, 6 and 8 seconds (s) and **Spatial Span (SS)** working memory test with the same delays. **Data analysis**. The data will be analysed for each illusion separately, with mixed-model Repeated Analysis of Variance with drug as a within-subject factor, sex of the participants and drug-order as between-subject factors, and with age and nicotine use (daily cigarette use) as covariates, if the data meet the assumptions required for ANOVA. If the data do not meet these assumptions, they will be analysed for drug induced differences in the frequency of responses with a bootstrap kernel density approach. All statistics will be calculated using R statistics and the "ez" Package for ANOVAs or the "sm" Package for kernel density analysis of frequencies on the measures taken during each of the illusions as the dependent variables of interest. Relationships between measures from the illusions and scales will be analysed. We will use a linear modelling approach for these analyses.

9. **Sampling**	We have conducted a power analysis using data from previous years and have found that the minimum sample size required is 26 (Cohen's F=.34) for each experiment. We have successfully recruited 24-32 volunteers per year in the previous 8 years.<br><br>If using a sample, explain how the size and profile of the sample to be recruited is adequate to answer the research question.

10. **Recruitment methods:**	Participants will be recruited by word of mouth, lecture announcements, by paper advertisements (campus noticeboards), social media or university-wide emails. Lecture announcements will be made during lectures by the supervisors in the Neuroscience and Pharmacology lectures. If interest is shown, a prospectus package is sent. The package will contain the PIF, PCF, nabilone consumer medication information and contact information. Attention will be drawn to the exclusion and inclusion criteria in the PIF.<br><br>Please attach copies of advertisements, flyers, posters, emails, etc.

11. **Data management and publication plan:**	All original data will be stored digitally wherever possible. If a physical form exists (such as in the case of written questionnaires), responses will be transformed into a digital medium. Data will be stored on a password protected computer. If physical data files are unable to be digitized, then they will be stored in the Pharmacology Department, M Block of QEII Medical Centre or to a university data storage system at a later date. All voice recordings will be deleted after 2 years in a non-recoverable way to ensure destruction of files.<br>All original data will be kept for 14 years. During the 14 years, data will be pooled in such a way so that personal identifiers will be removed, and only relevant information are kept. Pooled data will then be stored in a spreadsheet. After 14 years or until publication (whichever is longer) the data will then be irreversibly destroyed from all systems. Data directly used for publications may be kept on a password protected computer or be transferred to a storage medium (e.g., USB stick, CD-ROM) in case of corrections, retractions or meta-analyses. Data will not be stored online, except for published data available online. Data will be expected to be presented in several publications and conferences. Preliminary presentation of results at seminars and conferences may occur.<br><br>Research records must be retained for a minimum of 7 years after date of publication or project completion, whichever is the latter (Western Australian University Sector Disposal Authority).<br>How and where will you record, store, share, transmit, and archive your data? Discuss retention, security and data sharing plans. How will you publish / disseminate your work.<br>Consult the *Australian code for the responsible conduct of research*, section 2; and, the UWA Research Management Toolkit.

Ethics Themes: risk and benefit, consent

**12. Potential harms or risks to participants?**<br><br>NS §2.1	**Risks**<br>The use of any drug carries risks. Nabilone produces relaxation, drowsiness, and euphoria in the recommended dosage range. Other side effects may include vertigo, dry mouth, ataxia, headache, and concentration difficulties. Nevertheless, tolerance to these effects develops rapidly and is readily reversible. Nabilone may elevate supine and standing heart rates and cause supine and orthostatic hypotension. In clinical studies, oral administration of 2 mg of nabilone caused decrease in airway resistance in normal controls but had no effect in patients with asthma. No other nontherapeutic effects of clinical significance due to nabilone have been reported.<br><br>Nabilone appears to be completely absorbed from the human gastrointestinal tract when administered orally. Following oral administration of a 2 mg dose of radiolabeled nabilone, peak plasma concentrations of approximately 2 ng/mL nabilone and 10 ng equivalents/mL total radioactivity are achieved within 2.0 hours. The plasma half-life (T 1/2) values for nabilone and total radioactivity of identified and unidentified metabolites are about 2 and 35 hours, respectively. The initial rapid disappearance of radioactivity represents uptake and distribution of nabilone into tissue and the slower phase elimination by metabolism and excretion. The apparent volume of distribution of nabilone is about 12.5 L/kg. Nabilone exhibits dose linearity within its therapeutic range. Clinical data suggests that the intake of food does not significantly affect either the rate or extent of absorption.<br><br>The participants are provided with the drug information sheet for nabilone that describes the side effects and adverse effects. The risks are also clearly described in the Participant Information Sheet, and gone over during the informed consents process.<br><br>**Risk minimisation**<br>Risks are minimised by the following:<br>1) Exclusionary criteria include all known or suspected risk factors for adverse reactions to nabilone, and these are clearly described in the participant information sheet. Participants who have history of hypersensitivity to any cannabinoid will be excluded from the study.<br>2) The participants have an initial interview/examination by one of the psychiatrists (or one of their registrars) who are co-investigators. This includes a general medical and psychiatric examination, and serves to ensure that none of the exclusionary criteria are met.<br>3) Blood pressure and heart rate and regularity of the heartbeat are taken prior to administration of drug or placebo with three replicates. If the average blood pressure is >140/90 (hypertension), or <90/60 (hypotension), or the average heart rate is >100bpm, or if an arrhythmia is consistently detected on each replicate, the participant is thanks and excluded from the study.<br><br>Possible changes in mood and other adverse behavioural effects may occur in subjects receiving nabilone. Therefore, participants will remain under supervision of CI while using nabilone. Participants receiving nabilone treatment will be specifically warned not to drive, operate machinery, or engage in any hazardous activity while receiving nabilone. Also, participants receiving nabilone treatment would be advised to avoid alcohol, sedatives, hypnotics, or other psychoactive substances prior and post-treatment because these substances can potentiate the central nervous system effects of nabilone.<br><br>The Chief Investigator, or suitable proxy (Profs David Joyce or Fiona Pixley whose offices and laboratories are both near the testing lab) if CI is unavailable, will be present or nearby at all testing times. PhD research students will be present throughout the testing sessions. One of the psychiatrists is on call at all times. A direct call to the psychiatrist on call will be made immediately on their cell phone. There is a first aid kit in the next room and a defibrillator nearby. All personnel have been trained in First Aid, and in the use of the defibrillator. If the medical officer determine that a serious non-psychiatric medical condition has arisen, an ambulance will be called and will take the participant to the SCGH emergency, with the psychiatrist or the CI in attendance. To this point, we have not needed to manage any such event. The dose we use is similar to the daily dose of a chemotherapy patient receiving nabilone to treat vomiting and nausea and we don't anticipate serious adverse effects. Nonetheless, we are prepared for any such events.<br><br>See NS §2.1 - consider illness or injury, potential side effects; but also include potential embarrassment, economic loss, exposure to prosecution, anything stressful, noxious or unpleasant. Ensure you address these in your Participant Information Forms (PIF) if you are using those.<br>**Explain how this research justifies the burden and risks to participants?**

**13. Potential harms or risks to researchers?**	There are no more risks to the research team than those normally associated with spending time in the company of other human beings. Such commonplace risks are minimised by ensuring that experimenters are not left alone with the participants (e.g., female experimenter alone with male participant or vice versa). Each experimenter wears a duress alarm, the pressing of which will bring in a variety of medical and security people very quickly. A senior person (usually the CI) is always present in the next room, with a view of the test room through a one-way mirror, which is described to the participant before testing commences. There is a sign on the wall that indicates aggression towards others in not tolerated in the hospital. The test room has two exists, one door that opens one way and another that opens in the other direction such that no participants can "trap" an experimenter in the room or keep people out. Each door is equipped with a viewing portal. The experimenters are given some training in detecting potentially risky changes in behaviour. They are instructed to tell the participants they need to speak to their supervisor and leave the room to come talk with the supervisor if they detect such changes or feel uncomfortable for any reason.<br><br>Does this work open the research team to direct or indirect risk? Please explain.

**14. Will participants be given financial or non-financial incentives?**	Yes: ☐ ➜ No: ☑	NS §2.2.10-11 concerns inducements.
**Please describe:**	Click here to enter text.<br><br>NS §2.2.10 - payment that is disproportionate to the time involved, or any other inducement that is likely to encourage participants to take risks, is ethically unacceptable.	

**15. Will all participants provide consent?**	Yes: ☑ ➜ No: ☐ ➜	NS §2.2 concerns issues of consent.<br>NS §2.3 concerns potential waiver of consent.<br>*Attach Participant Information and Consent Forms.*
**How will you obtain consent, or justify a waiver?**	Participants will read through a participant information form that outlines relevant details about the research project. The information form will include information about treatments, groups and apparatus with enough detail to help them inform consent, but not enough to form biases or expectations. The form will also outline any risks involved. Participants will be then required to provide informed consent prior to commencement of the first experiment day.<br><br>Describe how you will deal with §2.2 and §2.3 of the *National Statement* regarding consent. If you request waiver of consent you must address NS §2.3. For a waiver, please also see the following section regarding the *Privacy Act*.	
**Privacy Act 1998, Sections §95 and §95A**	If you do not have written consent AND you are requesting a waiver of consent to access Commonwealth or private-sector data, you will need to justify how the public interest value of your research relevant to public health or public safety out-weigh the public interest in the protection of privacy. There are guidelines to assist you with this and you need to use them to make your case to the HREC. To use health information from a Commonwealth Government agency, see: Guidelines Under Section 95 of the Privacy Act 1988 available at: http://www.nhmrc.gov.au/_files_nhmrc/publications/attachments/e26.pdf To use health information from a private sector source, see: Guidelines approved under Section 95A of the Privacy Act 1988 available at: http://www.nhmrc.gov.au/_files_nhmrc/publications/attachments/e43.pdf	
	**Do you need health data from Commonwealth agencies (§95)?**	NO
	**Do you need health data from private-sector sources (§95A)?**	NO

**16. Will the research use deception, concealment or incomplete disclosure?**	Yes: ☐ ➜ No: ☑	NS §2.3 discusses use of deception, covert observation, concealment, and incomplete disclosure.
**Please describe:**	Click here to enter text.<br>Explain why this is essential to the research aims. How will participants be de-briefed after the experiment?	

Ethical considerations specific to research methods or fields

**17. Will you make video, photograph, or audio recordings?**	Yes: ☑ No: ☐ ↓	NS §3.1 provides guidance on recording.
**Please describe:**	Audio recordings will be taken for the Phantom Words Illusion test. Any other words besides the ones in which the test subjects are listening are to be spoken out loud and recorded using a voice recorder.<br><br>Address cultural issues if applicable. Ensure you explain storage of this material in your data management plan (above). Ensure you advise of this in your Participant Information and Consent forms.	

**18. Use of Qualitative Methods**	Yes: ☐ No: ☑ ↓	See special considerations of qualitative methods in NS §3.1
**Comments regarding** NS §3.1	Click here to enter text.	

**19. Use of data from data banks**	Yes: ☐ No: ☑ ↓	See special considerations of data banks in NS §3.2
**Comments regarding** NS §3.2	Click here to enter text.	

**20. Interventions, therapies, trials**	Yes: ☑ No: ☐ ↓	See special considerations of interventions, therapies, clinical and non-clinical trials in NS §3.3
**Comments regarding** NS §3.3	While this is considered a clinical trial, it is not a test of a new drug, nor does it have any financial implications or drug development or intellectual properties. We will be using the "drug challenge" model as a form of basic research to further understand the neurochemical and neurophysiological circuitry, and how the theorized dependency of psychoses on CB1 receptor activation is produced. Nabilone was chosen for the study due to its good record of safety. The dose of nabilone (2mg, taken orally, twice a day – the average clinical dose) used in this study has shown minimal risks.	

**21. Human Tissue**	Yes: ☑ No: ☐ ↓	See special considerations of tissue use in NS §3.4
**Comments regarding** NS §3.4	We will collect 1 saliva sample (approx. 1.0 – 2.0ml, but participants are encouraged to provide as much saliva as possible) per participant on the first testing day, prior to any drug administration so as to facilitate genetic analysis of each participant. This procedure is less invasive than blood sampling.<br><br>This includes collection of blood, tissue, bone, fluids, hair, teeth, DNA.<br>Include explanation of biobank use, and if samples are an ongoing part of the research, or merely to assess entry criteria.<br>If human bloods or tissue are used – please contact Biosafety Office	

22. Human Genetics	Yes: ☑ No: ☐	See special considerations of genetics in NS §3.5
Comments regarding NS §3.5	All saliva samples collected will be processed for characterization of catechol-O-methyltransferase (COMT) polymorphism (Biosafety will be conducted).<br>If Genetic manipulation is involved – please contact Biosafety Office	

Specific details of interventions, therapies and trials

23. Are drugs, biological agents or therapeutic devices to be used?	Yes: ☑ No: ☐	See the guidelines in NS §3.3<br>**Note:** A Clinical Trials Notification (CTN) or Clinical Trial Exemption (CTX) from Therapeutic Goods Administration (TGA) will be required if this is a clinical trial.
Attach full protocol	This is not a clinical trial in the sense of a test of new drug. That is, it is not a phase I, II, III or IV trial, nor is it a post-marketing trial.<br>*Attach a copy of the full protocol.*<br>*If you have a TGA CTN/CTX, please attach.*	

24. Will invasive procedures be used?	Yes: ☐ No: ☑	NS §3.3
Attach full protocol	*Attach a copy of the full protocol.*	

25. Will there be a placebo or non-treatment group?	Yes: ☑ No: ☐	NS §3.3 See 3.3.10 in particular.
Please describe:	Placebo control is in place, but as the study is a cross-over design, each participant acts as their own control, with half of the participants receiving placebo first and nabilone the second week, while the other half receive nabilone first and placebo the second week. This is the best comparator as we will be comparing the effects of nabilone on each participant's subjective state on drug and on placebo.<br>If a placebo is to be used, explain why it is the best comparator.	

26. Will (ionising) radiation be used?	Yes: ☐ No: ☑	Review the ARPANZA guidelines.<br>If Yes, you will also need to obtain approval from UWA Health and Safety for this work.
Please describe:	Click here to enter text.<br>Address radiological protection of participants involved exposed to x rays, CT-scan, DEXA, radiopharmaceuticals, fluoroscopy, radiation for cancer or benign lesions. Information at the Australian Radiation Protection and Nuclear Safety Agency (ARPANSA) will assist here.	

Does the research focus on any of the following groups of people?

27. Pregnant women, or Human ovum, embryo, or foetus	Yes: ☐ No: ☑	See special considerations for work with pregnant women, ova, embryo or foetus in NS §4.1
Comments regarding NS §4.1	Click here to enter text.	

**28. Children or young people (< 18 y.o.)**	Yes: ☐ No: ☑ ↓	See special considerations for work with young people in NS §4.2
**Comments regarding** NS §4.2	Participants must be over 18.	

**29. Dependent or unequal relationships**	Yes: ☑ No: ☐ ↓	See special considerations for work with people in dependent relationships in NS §4.3
**Comments regarding** NS §4.3	It is likely that some participants will be the CI's students in undergraduate or graduate levels. However, the CI is not actively recruiting other than announcing to classes that if they are interested in participating to contact the CI (as in the advertisement), in which case the CI passes the contact details on to the other experimenters to do the recruitment, as with any one else that contacts him from the advertisements. There is no motivation by the CI to offer inducements, as internally generated motivation for participating provides the best way of having participants come for both days of testing, whereas external inducements tend to be less effective motivators.<br><br>**Declare any relationship between the researcher and potential participants.** For example, private clients, relatives, colleagues, students of researcher. **Also any dependent relationships or perceived relationships with an organisation** which could influence participation or responses. How will you deal with these relationships and dependencies in your research design?	

**30. Highly dependent on medical care**	Yes: ☐ No: ☑ ↓	See special considerations for work people dependent on medical care in NS §4.4
**Comments regarding** NS §4.4	Click here to enter text.<br><br>**Also, declare any relationship between the researcher and potential participants.** How will you deal with these relationships and dependencies in your research design?	

**31. Cognitive impairment, intellectual disability, or mental illness**	Yes: ☐ No: ☑ ↓	See special considerations for work with people with cognitive impairment in NS §4.5
**Comments regarding** NS §4.5	Click here to enter text.<br><br>**Also, declare any relationship between the researcher and potential participants.** How will you deal with these relationships and dependencies in your research design?	

**32. Potential exposure of illegal activities**	Yes: ☑ No: ☐ ↓	See special considerations for work with potential exposure of illegal activities in NS §4.6
**Comments regarding** NS §4.6	We ask participants about their recent drug use so that we can exclude them if they have used any of a variety of drugs around the time of testing, and we also ask specifically about prior cannabinoid use, as this is important to know (i.e., they have had an adverse reaction to cannabinoids in the past, we will exclude them from the study). These data will not be recorded, but will be used to determine if exclusionary criteria are met.	

33. Aboriginal or Torres Strait Islander People	Yes: ☐ No: ☑	See special considerations for work with indigenous people in NS §4.7
Comments regarding NS §4.7	Research project does not research Aboriginal or Torres Strait Islander people. However, they may choose to participate in the experiment.	

34. Are any participants outside Australia?	Yes: ☐ No: ☑	NS §4.8 explains specifics of working outside Australia.
Countries and approvals:	Click here to enter text.<br>NS §4.8 Explain the ethical review process of the countries where overseas participants reside. Note: Australian ***and*** overseas ethics approval will probably be required.	

35. Is there a potential conflict of interest?	Yes: ☐ No: ☑	NS §5.4 addresses conflicts of interest in detail.
Please describe:	Click here to enter text.<br>For example: are there any private, personal, or financial connections between funder and researcher? **Actual or perceived? See:** NS§5.4.	

36. Other comments?	Click here to enter text.<br>Are there other issues you believe to be relevant to the ethical review of this research?

Attachments Checklist - Please attach the following if applicable:

**Ethics approval from non-UWA HREC**	☐
**Full medical research protocol and/or Student Research proposal**	☑
**Recruitment material**<br>(e.g. advertisements, posters, flyers)	☑
**Participant Information Form (PIF)**	☑
**Participant Consent Form (PCF)**	☑
**Additional PIF and PCF for parent, teacher, school, as needed**	☐
**Questionnaire / survey instrument**	☑
**Other docs** (e.g. contracts, agreements, focus group docs, detailed procedure info)	☐
**Translations, where languages other than English are used above.**	☐
**Related Approval & supporting documents**	☐
**TGA CTN acknowledgement letter**	☐
**If human bloods or tissue are used – please contact Biosafety Office**	☐

The following paragraph must be included in all **Participant Information Form (PIF) and Participant Consent Form (PCF)**:

"Approval to conduct this research has been provided by the University of Western Australia, in accordance with its ethics review and approval procedures. Any person considering participation in this research project, or agreeing to participate, may raise any questions or issues with the researchers at any time.

In addition, any person not satisfied with the response of researchers may raise ethics issues or concerns, and may make any complaints about this research project by contacting the Human Ethics Office at the University of Western Australia on (08) 6488 3703 or by emailing to humanethics@uwa.edu.au

All research participants are entitled to retain a copy of any Participant Information Form and/or Participant Consent Form relating to this research project."

Certification / signatures

<table>
<tr><td rowspan="2">Chief Investigator or Supervisor of Higher Degree Research Student:</td><td colspan="4">I declare that:
• The information provided in this application is truthful and as complete as possible.
• I undertake to conduct the research in accordance with the approved protocol, the National Statement on Ethical Conduct in Human Research, 2007, relevant legislation and the policies and procedures of The University of Western Australia.
• Where I am the Project Supervisor for research described to be conducted by a student of The University of Western Australia, I declare that I have provided guidance to the student in the design, methodology and consideration of ethical issues of the proposed research; that the student has received the relevant research and ethics training for this project; and, that I will monitor the project during data collection.
• I make this application on the basis that the information it contains is confidential and will be used by The University of Western Australia for the purposes of ethical review and monitoring of the research project described herein, and to satisfy reporting requirements to regulatory bodies. The information will not be used for any other purpose without my prior consent.

Signed:
(see comment below about using UWA email instead of ink signatures)</td></tr>
<tr><td>Name:</td><td>Click here to enter text.</td><td>Date:</td><td>Click here to enter a date.</td></tr>
</table>

<table>
<tr><td rowspan="2">Head of School Declaration:</td><td colspan="4">(Where the Head of School or nominee has a conflict of interest with the proposed research, e.g. an investigator on the project, a member of the research group, or a personal relationship to any member of the research team, this Declaration is to be completed by the Deputy Head of School.)

I declare that:
• I am satisfied that an adequate peer review has been conducted and that the research proposal is ready for submission for ethics approval.
• The resources required to undertake this project are available.
• The researchers have the skill and expertise to undertake this project appropriately.

Signed:
(see comment below about using UWA email instead of ink signatures)</td></tr>
<tr><td>Name:</td><td>Click here to enter text.</td><td>Date:</td><td>Click here to enter a date.</td></tr>
</table>

UWA policy deems this document as signed, if you send it attached to an email **from your UWA email address**. Alternatively you can sign the signature page, scan that page and send it with the application documents.

For example, the form can be filled out by the CI, emailed as an attachment to the Head of School, and then **Forward** (to ensure all attachments and email from/to/date lines are carried forward) to the Human Ethics office at humanethics@uwa.edu.au – this method avoids the need for ink signatures, preserves the best available viewing and search quality, saves paper and saves time.
