# Supplementary material for: The effects of caffeine and d-amphetamine on spatial span task in healthy participants
Source: PLoS One. 2023 Jul 13;18(7):e0287538. doi: 10.1371/journal.pone.0287538 (PMC10343048; doi:10.1371/journal.pone.0287538)
Supplement: S4 File — (PDF) [file pone.0287538.s004.pdf]

Human Ethics

Office of Research Enterprise

The University of Western Australia
M459, 35 Stirling Highway
Crawley WA 6009 Australia

T +61 8 6488 3703 / 4703
F +61 8 6488 8775
E humanethics@uwa.edu.au

CRICOS Provider Code: 00126G

Our Ref: RA/4/20/4558

29 June 2018

Professor Mathew Martin-Iverson
School of Biomedical Sciences
MBDP: M510

Dear Professor Martin-Iverson

HUMAN RESEARCH ETHICS APPROVAL - THE UNIVERSITY OF WESTERN AUSTRALIA

Nabilone and Caffeine Effects on the Perceptions of Visually, Auditory, Tactile and Multimodal Illusions in Healthy Volunteers

Ethics approval for the above project has been granted in accordance with the requirements of the *National Statement on Ethical Conduct in Human Research* (National Statement) and the policies and procedures of The University of Western Australia. Please note that the period of ethics approval for this project is five (5) years from the date of this notification. However, ethics approval is conditional upon the submission of satisfactory progress reports by the designated renewal date. Therefore initial approval has been granted from 29 June 2018 to 28 June 2019.

You are reminded of the following requirements:

1. The application and all supporting documentation form the basis of the ethics approval and you must not depart from the research protocol that has been approved.
2. The Human Ethics office must be approached for approval in advance for any requested amendments to the approved research protocol.
3. The Chief Investigator is required to report immediately to the Human Ethics office any adverse or unexpected event or any other event that may impact on the ethics approval for the project.
4. The Chief Investigator must submit a final report upon project completion, even if a research project is discontinued before the anticipated date of completion.

Any conditions of ethics approval that have been imposed are listed below:

Special Conditions

None specified

The University of Western Australia is bound by the *National Statement* to monitor the progress of all approved projects until completion to ensure continued compliance with ethical principles.

The Human Ethics office will forward a request for a Progress Report approximately 30 days before the due date.

If you have any queries please contact the Human Ethics office at humanethics@uwa.edu.au.

Please ensure that you quote the file reference – RA/4/20/4558 – and the associated project title in all future correspondence.

Yours sincerely

Mark Davies

Manager, Human Ethics

Name	***Faculty / School***	***Role***
Professor Mathew Martin-Iverson	School of Biomedical Sciences	Chief Investigator
Associate Professor Jennifer Rodger	School of Biological Sciences	Co-Investigator
Professor Joseph Lee	Medical School	Co-Investigator
Dr Emily Hepple	North Metropolitan Health Service	Co-Investigator
Dr Rajan Lyyalol	North Metropolitan Health Service	Co-Investigator

Student(s): Jit Lim, Faiz Kassim, Fui Voon, Benjamin Peters, Aakanksha Sharma
